# Aggregation-Driven Photoinduced α-C(sp^3^)–H Bond Hydroxylation/C(sp^3^)–C(sp^3^) Coupling of Boron Dipyrromethene Dye in Water Reported by Near-Infrared Emission

**DOI:** 10.1021/jacs.4c02019

**Published:** 2024-05-31

**Authors:** Adelajda Shahu, Vasilis Petropoulos, Emmanuel Saridakis, Vyron S. Petrakis, Nikolaos Ioannidis, George Mitrikas, Andriana Schiza, Christos L. Chochos, Eleni-Marina Kasimati, Anastasia Soultati, Maria Christina Nika, Nikolaos S. Thomaidis, Mihalis Fakis, Margherita Maiuri, Giulio Cerullo, George Pistolis

**Affiliations:** † Department of Chemistry, National and Kapodistrian University of Athens, Athens 15771, Greece; ‡ Institute of Nanoscience & Nanotechnology, NCSR “Demokritos”, Athens 15310, Greece; § Institute of Chemical Biology, National Hellenic Research Foundation, Athens 11635, Greece; ∥ Department of Physics, University of Patras, Patras 26504, Greece; ⊥ Department of Physics, Politecnico di Milano, Milano 20133, Italy

## Abstract

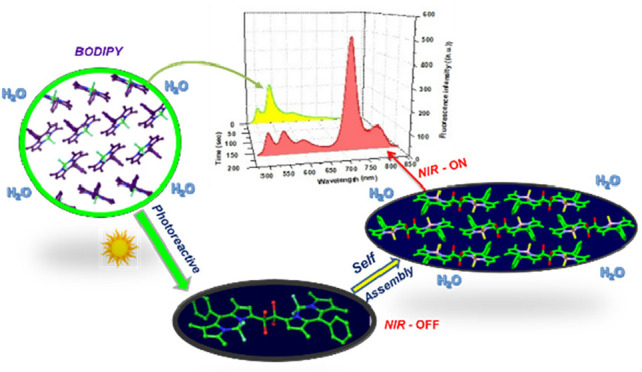

Molecular aggregation is a powerful tool for tuning advanced materials’ photophysical and electronic properties. Here we present a novel potential for the aqueous-solvated aggregated state of boron dipyrromethene (BODIPY) to facilitate phototransformations otherwise achievable only under harsh chemical conditions. We show that the photoinduced symmetry-breaking charge separation state can itself initiate catalyst-free redox chemistry, leading to selective α-C(sp^3^)–H bond activation/C_sp^3^_–C_sp^3^_ coupling on the BODIPY backbone. The photoproduction progress was tracked by monitoring the evolution of the strong Stokes-shifted near-infrared emission, resulting from selective self-assembly of the terminal heterodimeric photoproduct into well-ordered J-aggregates, as revealed by X-ray structural analysis. These findings provide a facile and green route to further explore the promising frontier of packing-triggered selective photoconversions via supramolecular engineering.

Multichromophoric aggregates,^1−4^ formed either by self-assembly or by covalently bridged dyes, have led to the emergence of new and useful packing-induced optical properties and functions not present in isolated dyes, such as fluorescence,^1,5−7^ intermolecular charge transfer,^8−10^ singlet fission,^11^ aggregation-induced emission^12^ (AIE), reactive oxygen species (ROS) generation,^13^ etc. In recent years, the photoinduced symmetry-breaking charge separated (SB-CS) state has attracted enormous interest from both biological^14^ and materials science perspectives.^15^ However, most studies have focused on dimeric and oligomeric constructs of similar dyes,^11,15,16^ which suffer from short-lived SB-CS states.

Extended molecular aggregates, especially those with crystalline order, are promising candidates for the efficient production of long-lived SB-CS states. Such highly ordered packing motifs decisively affect the energy band levels and the extent of excited state delocalization/dynamics.^1,2,17−19^ Besides, they should be conducive to initiating redox chemistry on diffusion-controlled time scales. For instance, distinctive packing patterns of some common dyes^20−25^ have manifested themselves as efficient photocatalysts for hydrogen production,^21−23^ oxygen evolution,^20^ solar fuels,^25^ and other organic transformations.^24^ Boron dipyrromethene (BODIPY) dyes^26−32^ (Figure 1) are known for their excellent photophysical and photosensitizing properties, placing them at the forefront of current research.^33−35^ Recently, J-aggregates formed by self-assembling some BODIPY dyes are valued for their ability to manifest extraordinary AIE tunability in the visible and NIR regions,^36−43^ with broad prospects for clinical applications.^44,45^ Surprisingly, however, aggregation-driven photochemically activated transformations at the BODIPY backbone itself remain unexplored.

**Figure 1:**
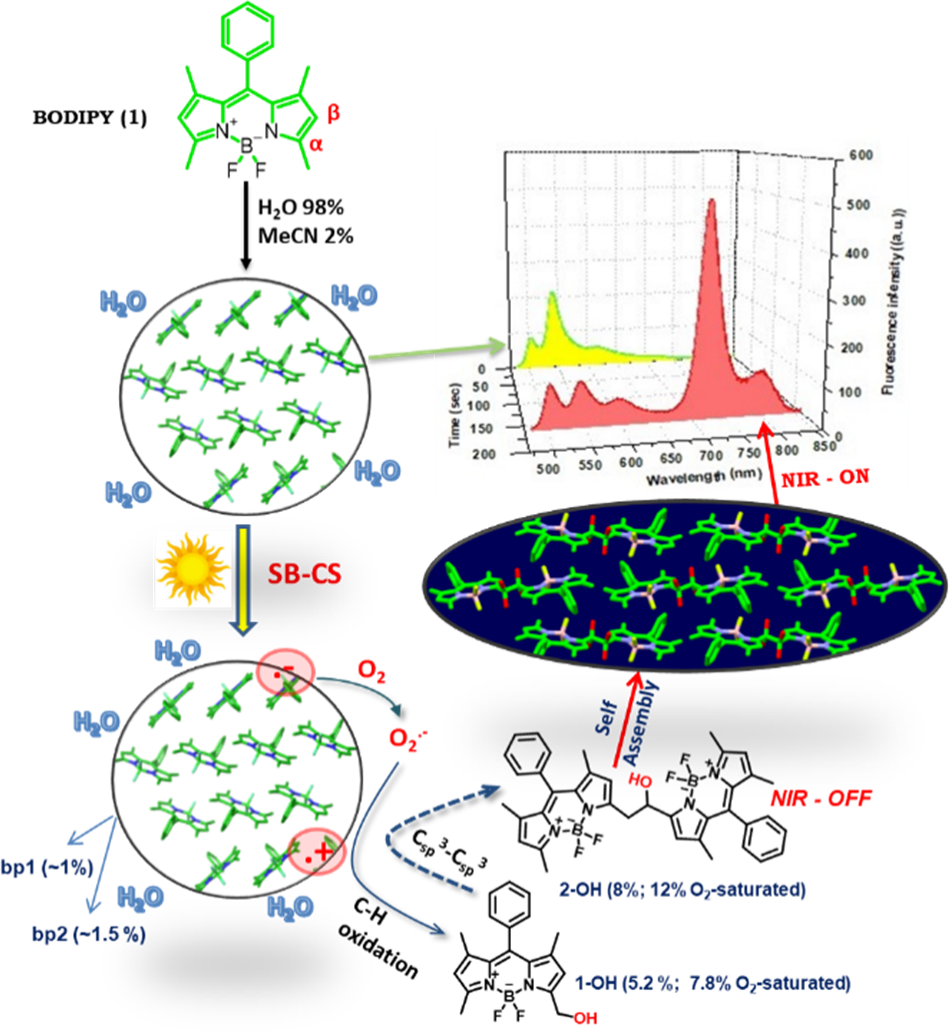
Schematic illustration of aggregation-triggered photoinduced α-C_sp_^3^–H bond hydroxylation and C_sp_^3^–C_sp_^3^ bond coupling. NIR-AIE of aggregated **2-OH** (red spectrum) versus that of **1**’s aggregates before illumination (yellow).

Here we report a clear departure from the known photochemical stability of the BODIPY framework, upon nanocrystalline aggregation in water, according to the scheme presented in Figure 1. We demonstrate that the SB-CS state itself, rather than the commonly occurring mediating triplet-state,^12,13,44^ initiates redox chemistry involving ROS, leading to α-C(sp^3^)–H bond hydroxylation followed by heterodimerization via C_sp^3^_–C_sp^3^_ coupling. Almost any source of visible light, including ambient sunlight, can be used to carry out this challenging class of reactions on the BODIPY scaffold, which are otherwise only possible using metal-containing catalysts and strong oxidants.^5,6,46−52^ Finally, the progress of photoproduction was tracked by monitoring the NIR signal resulting from the selective self-assembly of the terminal photoproduct **2-OH**.

The regular and widely investigated BODIPY **1** forms aggregates in aqueous solutions with high water fraction (f_w_ ≥ 95%) (Supporting Information (SI) page S4; Figure S1). The absorption and fluorescence spectra of **1** in an H_2_O/MeCN mixture (f_w_ = 98%) are significantly affected compared to those in MeCN, displaying well-known features associated with aggregation such as broadening, red-shift, low molar absorptivity (ε), reduced fluorescence yield (Φ_f_) and multiexponential fluorescence decay (Figures 2a, S2). The average hydrodynamic radius of the aggregates was measured to be 164 ± 4 nm by dynamic light scattering (DLS) (Figure 2a; inset).

**Figure 2:**
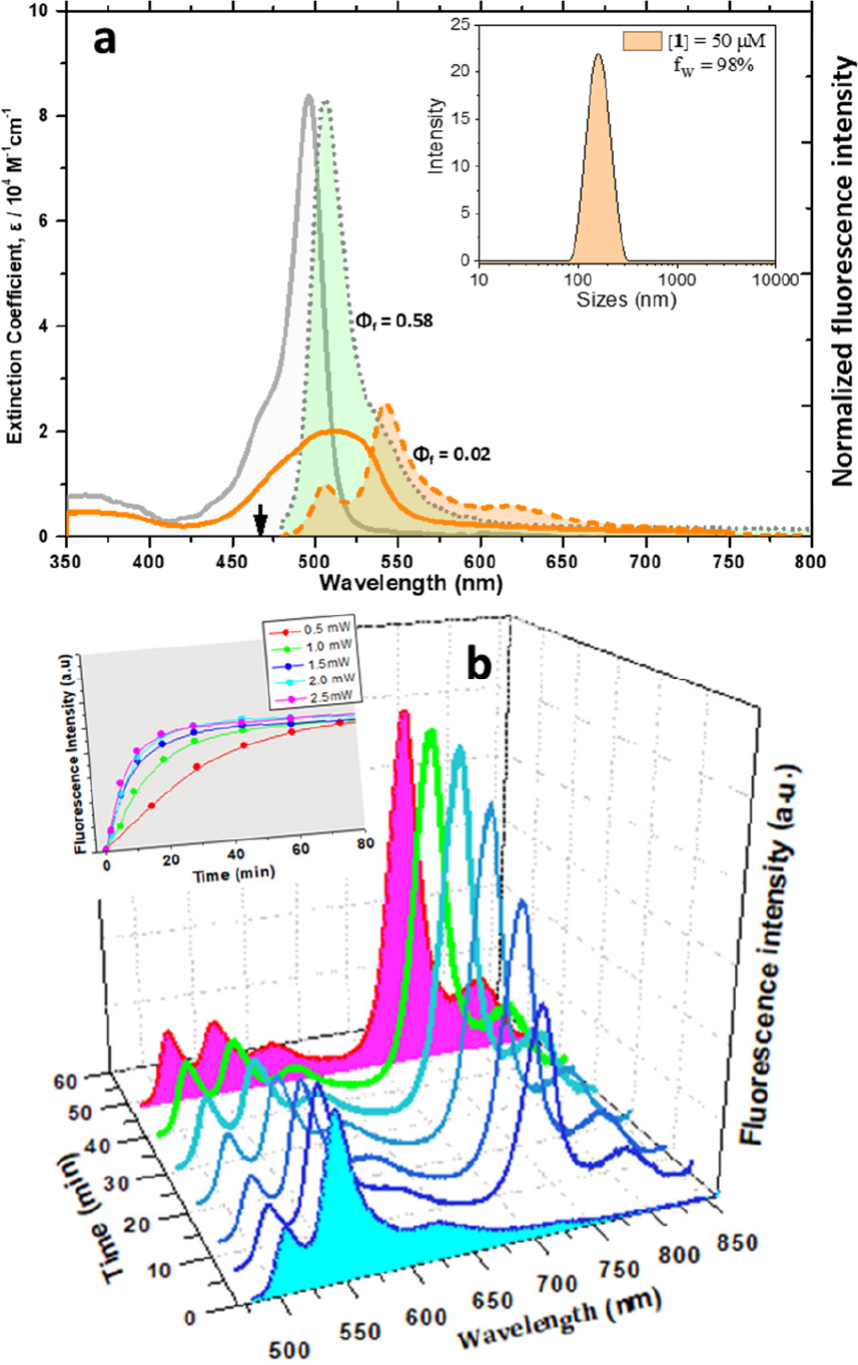
(a) Absorption and fluorescence spectra (exc: 470 nm) of **1** (50.0 μM) in MeCN and H_2_O/MeCN (f_w_ = 98%) in the dark; *inset*: DLS spectrum of aggregates. (b) Evolution of NIR emission spectra vs illumination time (1 mW white light LED); *inset*: time-dependence of NIR intensity at different LED powers (SI page S3).

The scanning electron microscopy (SEM) photomicrograph shows various sizes of plate-shaped crystalline aggregates ranging from ∼0.17 to 0.87 μm (Figure S3). In addition, their X-ray diffraction (XRD) pattern perfectly matches that of single crystals of **1** (Figure S4), where a J-type arrangement of adjacent units was manifested.^5^

When a solution of aggregates is exposed to visible light, new NIR emission features with high Stokes shift appear (700–850 nm; Figures 2b, S5), the intensity-rise of which becomes steeper and flattens out sooner as the power of the light source increases (Figure 2b; inset). To test whether light-induced transformations caused the emission, aqueous solutions of **1** were irradiated with visible light and the solid residue was analyzed by column chromatography (SI page S3). Two main hydroxylated photoproducts were detected, namely, **1-OH** (yield 5.6%) resulting from selective hydroxylation of the α-C(sp^3^)–H bond of **1** and the *α–α*-bridged heterodimer, **2-OH** (yield 8.2%) formed via **1-OH/1***α–α* C_sp_^3^–C_sp_^3^ coupling (Figure 1). The oxidative hydroxylation occurs exclusively and selectively at the α-methyl group position, and no β-hydroxy products were observed. We were also able to detect two minor side reaction products, **bp1** (a β- oxidized derivative of **1**) and **bp2** (an *α–β* ethylene bridged BODIPY dimer) in very low yields (∼1% and ∼1.5% respectively) together with an intractable mixture of polar decomposition products (SI pages S11–25).

Next, we screened the reaction conditions; first, when aggregation is suppressed by decreasing f_w_ < 80%, illumination has almost no effect on the solute (SI page S4). Second, O_2_ removal by degassing dramatically reduces the yield of hydroxylated photoproducts (<1%), whereas O_2_ saturation improves their yield by ∼50% (7.8% **1-OH** and 12% **2-OH**) compared to normal aerobic conditions. Third, when crystalline aggregates are removed from the aqueous phase by ultracentrifugation, their illumination has no effect. Fourth, shielding the solution from light completely inhibits the reactions. Following illumination of a sample containing the radical trapping reagent 5,5-Dimethyl-1-Pyrroline-N-Oxide (DMPO), the collected electron paramagnetic resonance (EPR) spectrum comprises two radical species (Figure 3b). The main four-line spectrum (*) shows an intensity ratio of 1:2:2:1 and a hyperfine coupling constant a_N_ = a_Hβ_ = 14.9 G assigned to the DMPO/OH• adduct.^53^ The minor species (▼) represents probably a degraded DMPO unknown species with a_N_ = 14.7 G. No signal attributable to the DMPO/OH• adduct was detected under argon atmosphere (Figure 3c and page S26), implying that water oxidation could not be the source of OH•. Yet, the involvement of singlet oxygen (^1^O_2_) in the DMPO/OH• adduct formation^54,55^ was excluded by several control experiments listed below: Phosphorescence of ^1^O_2_ at ∼1270 nm was not observed after pumping a sample of aggregates at 510 nm. The presence of NaN_3_, a well-known ^1^O_2_ quencher, did not suppress the yields of **1-OH** and **2-OH**. No EPR signal was detected when 2,2,6,6- tetramethylpiperidine (TEMP), a selective spin-trapping agent for ^1^O_2_, was used (Figure S30). These findings collectively point to a rapid transformation of an initially formed DMPO/O_2_^•–^ adduct to the observed stable DMPO/OH•.^56^

**Figure 3:**
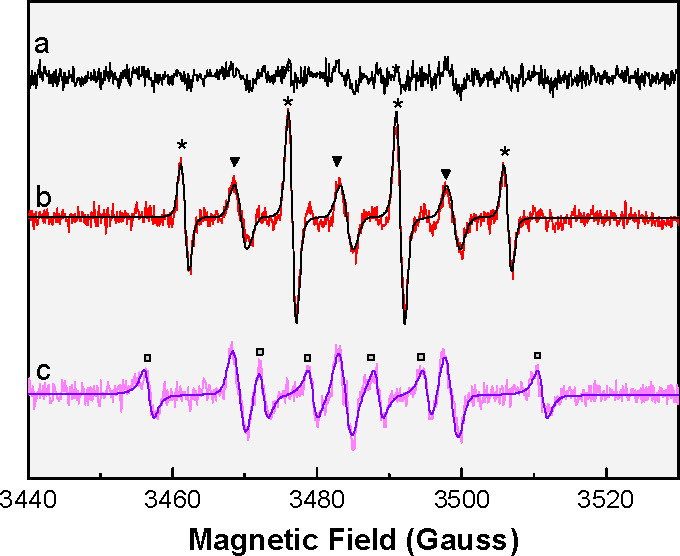
EPR spectra of aggregates ([**1**] = 50 μM) in the presence of 50 mM DMPO (a) before and (b) after illumination and (c) after degassing and illumination. Black lines represent spectral fits (b, c).

The potential of aggregates to undergo SB-CS was investigated by femtosecond transient absorption (fs-TA) spectroscopy. In MeCN and below the aggregation threshold (f_w_ ≤ 80%), the spectra show the typical decay of the bright excited state to the ground state (Figures 4a, b; S31–33). Upon aggregation, a new weak excited state absorption (ESA) band around 540 nm is observed, which grows with 3 ps lifetime (Figures 4c, d; S34–37). This band has been assigned to a radical anion associated with the formation of a SB-CS state in BODIPY pairs.^16^ The cation absorption feature is expected at ∼500 nm for **1**(57) and is masked by the ground state bleaching (GSB). The SB-CS state was found to possess multiexponential decay (<τ> ≈ 2.6 ns) and a long-lived (>50 ns) plateau using ns-TA (Figures S35–37; Tables S1–2). No evidence was found for the triplet manifold population,^58^ whose ESA signature is expected^59−61^ at ∼600 nm. Cyclic voltammetry confirms the exergonic CS process in S1, exhibiting an electrochemical gap of 0.71 V, which is well below the optical band gap (E_00_ = 2.25 eV) of aggregates (Figures S38–40).

**Figure 4:**
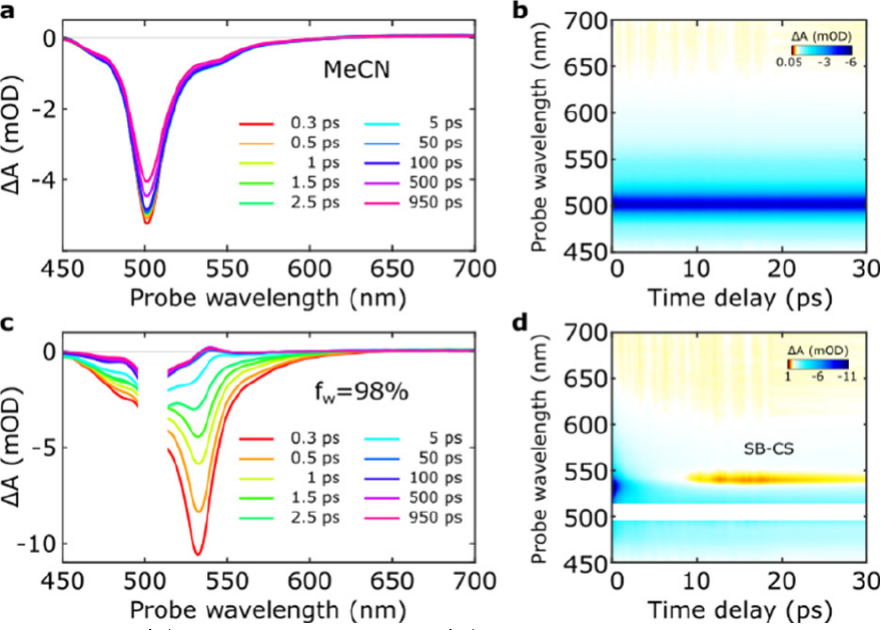
(a) fs-TA spectra and (b) 2D-plots for **1** in MeCN and (c), (d) of aggregates of **1** (f_w_ = 98%; [1] = 30 μM) respectively; exc: 500 nm.

We visualize the reaction sequence as follows (Scheme S1): (a) formation of O_2_^•–^ by interfacial electron transfer from the SB-CS state of aggregates of **1** to molecular O_2_, either directly and/or by interference of water radical anions^62^ (H_2_O^•–^). (b) the photogenerated radical cationic state at the C–H site of the α-CH_3_ group of **1** (whose acidity can be significantly altered in the radical cation state;^48,63^ p*K*_a_ < −10) can be transformed to stable neutral radical by proton transfer to water, considering the exergonic nature of the reaction.^48,64^ (c) O_2_^•–^ can react with the cationic state of the α- C–H site of **1** either directly due to its strong nucleophilic character^65^ or (d) because of the strong Brønsted basicity of O_2_^•–^, it can be protonated by H_2_O even in weakly acidic solutions^65,66^ to form the hydroperoxide radical^65^ HO_2_^•^ which can subsequently react with the stable α-centered neutral radical of **1** to form the hydroperoxide derivative. The latter being unstable (the O–O bond strength 34–45 kcal/mol of hydroperoxides is very weak), undergoes thermodynamically favored water-assisted cleavage to afford **1-OH**.^48,67^ (e) the latter can promote association with a closely spaced unit of **1** in J-aggregates to form the heterodimer, **2-OH**, via *α–α* C_sp_^3^–C_sp_^3^ oxidative coupling.

The NIR-AIE signal was further evaluated as a “reporter” to monitor the progress of photoproduction. Independent experiments demonstrated that it originates from the selective self-assembly of the terminal photoproduct **2-OH**, as explained below. Compared to its monomeric precursors **(1** and **1-OH)**, the highly coplanar structure of **2-OH** (Figure 5a) exhibits narrowed and bathochromically shifted absorption and emission spectral features in hexane (Figure 5b), suggesting J-type excitonic coupling between the bridged BODIPY dipoles^2^ (page S33). In aqueous solutions, the nonfluorescent monomer units of **2-OH** aggregate completely when f_w_ ≥ 80% (Figure 5c). Aggregation is accompanied by the appearance of a strongly Stokes-shifted (∼5000 cm^–1^) NIR emission spectrum (Φ_f_^NIR^ = 0.12), whose excitation spectrum matches that of the absorption of aggregates (Figure 5c; inset). The concentration-dependent absorption and emission spectra of **2-OH**, (0.05 ≤ [2-OH] ≤ 2.0 μM) are shown in Figure 5d and 5e, respectively. As seen, the ratio (A_518_/A_486_), the ratio R = (I_721_/I_548_) between the NIR intensity at 721 nm and that of the local excited-state (548 nm), as well as the Φ_f_^NIR^, soon level off above 0.5 μM (Figure 5d, 5e insets). Further increasing the **2-OH** concentration up to 20 μM has no effect on the absorption spectra (ε vs [2-OH]; Figure 5f); yet the intensity of the NIR emission increases linearly with concentration, highlighting its role as a ’reporter’ in monitoring **2-OH** photoproduction. Furthermore, the emission lifetime remains unaffected, indicating no changes in the intrinsic properties of the aggregates. (Figure S42). DLS analysis shows a narrow size distribution of particles peaking at 125 nm and remaining invariant above 2.0 μM. (Figure 5f inset). Consistent with DLS, AFM revealed elongated plates 30–250 nm in size with a peak at 125 nm and a thickness of ∼10 nm. (Figure 5g). These results indicate a highly favored self-assembly of **2-OH** units that evolves over a narrow and low concentration range (0.05–0.5 μM), resulting in homogeneous single-type assemblies. Single crystals grown from an aqueous solution of **2-OH** further confirmed the potential for a packing-mediated, water-stabilized low-energy emitting excited state.^1,37^ The dimeric units are packed (*b*-axis) in a linear arrangement forming ordered, quasi-one-dimensional layers, in which the J-dimers themselves are organized in a slip-stacked (J-type) mode (Figure 5h; SI page S33).

**Figure 5:**
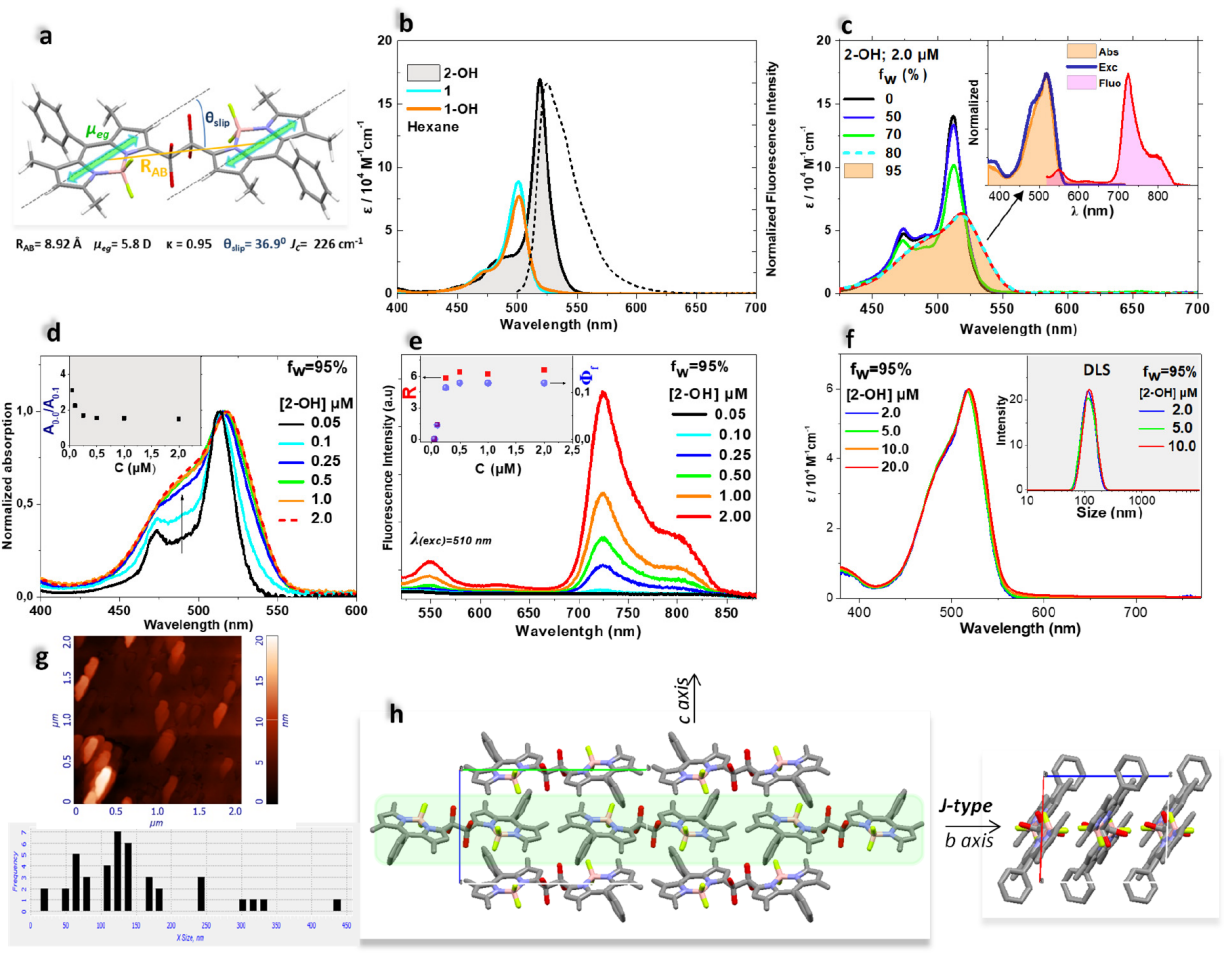
(a) X-ray crystal structure of **2-OH** obtained from hexane. The four alternative OH positions are due to the racemic mixture and crystal packing disorder. R_AB_ is the center-to-center distance between BODIPY rings; *θ*_*slip*_ stands for the angle formed by the transition dipole moments (μ_eg_) and κ is the orientation factor (eq S4); *J*_*c*_ is the calculated energy coupling. (b) Absorption spectra of **1**, **1-OH** and **2-OH** and fluorescence spectrum (dotted line; exc: 470 nm) in hexane. (c) Absorption spectra of **2-OH** in MeCN and H_2_O/MeCN mixtures with varied f_w_; *inset*: normalized absorption, fluorescence (exc. 510 nm) and fluorescence–excitation (em. 740 nm) spectra of aggregates of **2-OH**. (d) Normalized concentration-dependent absorption spectra of **2-OH**; *inset*: A_0–0_/A_0–1_ vs [2-OH]. (e) Concentration-dependent fluorescence spectra as in (d); *inset*: Φ_f_^NIR^ and R = I_NIR/_/I_LE_ vs [2-OH]. (f) Absorption and DLS (inset) spectra vs [2-OH]. (g) AFM photograph of crystalline aggregates and distribution of aggregates sizes (lower panel). (h) Long-range order in **2-OH** crystals grown from aqueous solution.

Further investigation revealed that J-aggregates of common, differently substituted BODIPY derivatives also exhibit similar photoconversions associated with their NIR-AIE fingerprints demonstrating BODIPY’s J-aggregates’ broad potential as photoreactive platforms. (Figure S43). These findings pave the way for engineering the performance of photo(re)active molecular packings by fine-tuning the *structure-packing-energy landscape* relationships. Furthermore, the unique features of in situ generated NIR-AIE such as, tunability, sharpness, biocompatibility, high luminescence, could benefit applications including high-fidelity bioimaging, frequency-controlled lighting, lasing etc.

## Supporting Information Available

Chemical procedures and methods, NMR and HRMS spectra, CV graphs, additional steady-state and transient spectroscopic data, X-ray diffraction, SEM images and EPR spectra (PDF)
