# Supplementary material for: Aggregation-Driven Photoinduced α-C(sp3)–H Bond Hydroxylation/C(sp3)–C(sp3) Coupling of Boron Dipyrromethene Dye in Water Reported by Near-Infrared Emission
Source: J Am Chem Soc. 2024 May 31;146(23):15659–65. doi: 10.1021/jacs.4c02019 (PMC11190975; doi:10.1021/jacs.4c02019)
Supplement: Supplementary file 1 — ja4c02019_si_001.pdf [file ja4c02019_si_001.pdf]

Supporting Information

Aggregation-Driven Photoinduced α-C(sp_3)-H Bond Hydroxylation / C(sp^3)- C(sp^3) Coupling of Boron Dipyrromethene Dye in Water Reported by Near-Infrared Emission

Adelajda Shahu[ab], Vasilis Petropoulos[de], Emmanuel Saridakis[b], Vyron S. Petrakis[ab], Nikolaos Ioannidis[b], George Mitrikas[b], Andriana Schiza[ac], Christos L. Chochos[c], Eleni-Marina Kasimati[b], Anastasia Soultati[b], Maria Christina Nika[a], Nikolaos S. Thomaidis[a], Mihalis Fakis[d], Margherita Maiuri[e], Giulio Cerullo[e] and George Pistolis[b]*

[a] *Department of Chemistry, National and Kapodistrian University of Athens, Athens, 15771, Greece.*
[b] *Institute of Nanoscience & Nanotechnology, NCSR "Demokritos, Athens, 15310, Greece.*
[c]*Institute of Chemical Biology, National Hellenic Research Foundation, Athens 11635, Greece*
[d]*Department of Physics, University of Patras, Patras, 26504, Greece.*
[e]*Department of Physics, Politecnico di Milano, Milano, 20133, Italy.*

Table of Contents **page**

Materials, General procedures for photochemical processes and Methods **S3 – S7**

Concentration-dependent absorption and fluorescence spectra of 1 **S8** **Fluorescence decays of 1 in various solvents and in H2O/MeCN (98/2 v/v)** **S8**

SEM photograph of 1 **S9**

FXRD of crystalline aggregates of 1 **S9**

Fluorescence-excitation spectrum of aggregates of 1 **S10**

1D and 2D NMR Characterization and HRMS spectra of 1-OH **S11 – S14**

1D and 2D NMR Characterization and HRMS spectra of 2-OH **S15 – S18**

1D and 2D NMR Characterization and HRMS spectra of bp1 **S19 – S21**

1D and 2D NMR Characterization and HRMS spectra of bp2 **S22 – S25**

Electron Paramagnetic Resonance (EPR) **S26**

Femtosecond and nanosecond Transient Absorption Spectroscopy **S27 – S30**

Cyclic voltammetry **S31**

Normalized absorption and fluorescence spectra of a crystalline film of 1 **S32**

Proposed Mechanism **S32**

Crystal Structure **S33**

Calculation of excitonic coupling **S34**

Nanosecond fluorescence dynamics of 2-OH (f_w=95%) versus concentration **S35**

Universality of phototransformations **S36**

References **S37 - S38**

Materials - General procedure for photochemical processes - Methods

Materials. All materials were purchased from commercial sources and used as received unless otherwise stated. All solvents were purchased as HPLC grade solvents. The starting materials, 1,3,5,7-tetramethyl-8-phenyl-4,4-difluoroboradiazaindacene (BODIPY **1**), 4,4-difluoro-8-(3, 5-di-tert-butylphenyl)-1,3,5,7-tetramethyl-4-bora-3a,4a-diaza-s-indacene (BODIPY **2**), 4,4-difluoro-8-(4-iodophenyl)-1,3,5,7-tetramethyl-4-bora-3a,4a-diaza-s-indacene (BODIPY **3**), 2,6-Diethyl-8-phenyl-1,3,5,7-tetramethyl-4,4-difluoro-4-bora-3a,4a-diaza-s-indacene (BODIPY **4**) were synthesized following published procedures.[1-4] BODIPY **5** was purchased commercially from Sigma-Aldrich.

General Procedure for Photochemical Processes. To a solution of **1** (8.1 mg, 0.025 mmol) in ACN (12.5 mL) was added distilled water to a final volume of 500 mL, under vigorous stirring at room temperature. A Xenon lamp (500 W) was focused at the center of a 500-mL reaction vessel containing the sample solution for internal irradiation. The sample solution was irradiated for 40 min under stirring and then the solvent was removed by rotary evaporation. The above procedure was repeated eight times to obtain a total amount of 64.8 mg of crude product. The solid residue was firstly separated by column chromatography using n-hexane/CH_2Cl_2 1:1, followed by 1:2, 1:4, and pure CH_2Cl_2. First, the residual educt was eluted as an orange-yellow fraction of greenish-yellow fluorescence (45 mg, 70% of unreacted starting material (BODIPY **1**) was recovered and used for another irradiation). The byproduct **bp2** (2 mg, 1.5 %) was eluted first, as a pink fraction of orange-yellow fluorescence, followed by the second byproduct **bp1** (0,7 mg, ~1 %), as a green-yellow fraction of green fluorescence. Subsequently, the main photoproduct, **2-OH** (10,9 mg, 8.2 %), was eluted as a deep red fraction of green-orange fluorescence, followed finally by a fourth product**,** **1-OH** (3,8 mg, 5.6 %), as a red-brown fraction of green-orange fluorescence. Each photoproduct was further purified by preparative thin layer chromatography (PLC, Silica gel 60 F254, 0.5 mm) using CH_2Cl_2 as the developing solvent.

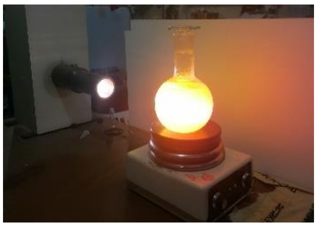

LED Irradiation: For the spectroscopic measurements, the samples were irradiated with a commercial white light LED (Thorlabs), either at a constant power (1 mW; Figure 2b) or at a variable power (0.5 - 2.5 mW; Figure 2b inset; main manuscript). The LED spectrum shown in the figure (grey color) covered the range 400-750 nm and was focused to a circular spot size of 1 cm^2, illuminating 3 ml of the sample placed in a 1 cm x 1 cm x 3 cm quartz cuvette. Throughout the illumination process, the sample was shielded from external light sources and

stirring was used to ensure uniformity. No evolution of the emission spectrum was observed when a solution of aggregates was placed in the dark, or when non-resonant excitation, no matching the absorption transitions of aggregated BODIPY **1** in 98% of water (orange spectrum), was used. This indicates that the aggregated state of BODIPY **1**, in its excited state, initiates the reaction mechanisms. Importantly, the LED intensities used (0.5 - 2.5 mW/cm^2) were an order of magnitude lower than sunlight sources. In fact, exposing a sample to daylight or even ambient laboratory light dramatically accelerated the time dependent spectral evolution.

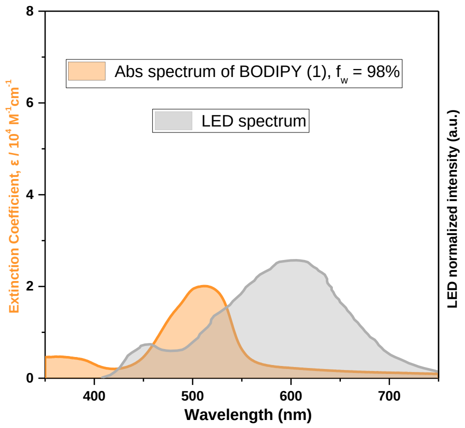

LED spectrum (grey color), used to monitor the photo-induced chemistry of **BODIPY 1**, overlapping the absorption spectrum of **BODIPY 1** in 98% of water (orange color).

Oxygen Effect: To clarify the role of atmospheric O2 on the photochemical reactions and to further increase the yields of hydroxylated compounds, i.e**.** **1-OH** and **2-OH**, we performed a new experiment using a solution of BODIPY **1** saturated with O2. More specifically, to a solution of BODIPY **1** (8.1 mg, 0.025 mmol) in ACN (12.5 mL) was added distilled water to a final volume of 500 mL, under vigorous stirring and then the solution was saturated with oxygen by bubbling with a balloon filled with O2 for 10 min in the dark. The solution was then irradiated with a 500 W Xenon lamp for 40 minutes while stirring and bubbling continued. After this step, the procedure was continued as described above. An increase of ~ 50% in the yields of **1-OH** and **2-OH** by ~ 50% was observed, giving a total yield of ~20% for hydroxylated compounds (7.8% **1-OH**; 12% **2-OH**) compared to 13.2% (5%; **1-OH** and 8.2% **2-OH**) under normal aerobic conditions.

The Role of Water: A solution of BODIPY **1** in water was prepared in the dark as described above. After ultracentrifugation of the sample, the crystalline aggregates were removed from the aqueous phase and were exposed to a 500 W Xenon lamp for several hours. There was no photochemical effect. This suggests that solvation of the aggregates is critical in triggering ROS formation and subsequent reactions. It has been observed that interfacial water plays an important role in surface reactions whose electrochemical performance can be significantly influenced (ref. 59 in MS) This is understood by the strong electric fields generated by (i) highly oriented water molecules towards the surface and (ii) spontaneous electron transfer leading to a water radical pair. The above imply that in the present work, the key initial step, namely the reduction of O_2 to superoxide anion ($O_2^{\cdot-}$), may not occur, fully or even partially, by direct electron transfer from the charge separated state of the photoexcited substrate (viz, anionic radical of BODIPY), but by the interference of hydrated electrons, i.e. water radical anions ($H_2O^{\bullet-}$); i.e., (BODIPY $^{\bullet+}$.......BODIPY $^{\bullet-}$) -e → $H_2O^{\bullet-}$.... O_2→ H_2O ... $O_2^{\cdot-}$

When nanocrystals are dispersed in water-rich solutions (typically fw > 90%), their hydrophobically-driven integrity is indeed maintained and they become photoreactive due to solvation. In contrast, in organic solvents, where BODIPYs are usually highly soluble, the nanocrystalline aggregates disintegrate in favor of monomeric

units. The presence of mere traces of water, on the contrary, does not appear to cause any photochemical reaction. For instance, concentrated stock solutions of BODIPY (e.g. 5.0 mM) in MeCN or THF containing traces of water have been found to be stable for several weeks after exposure to daylight.

Concentration Effect: Concentration-dependent experiments showed that 2 - 5 x 10^{-5} M is the most suitable concentration range to obtain homogeneously dispersed nanocrystals of BODIPY **1** in aqueous solutions when the molar fraction of water (f_w) is kept ≥ 95%, in agreement with related studies from the literature[5] (see also Figure S1). Attempts to use higher concentrations of **1** in the presence of water failed due to limited solubility, resulting in the agglomeration of dispersed nanocrystals into large precipitating crystals. Alternatively, we prepared highly concentrated solutions of 1 (up to 8 millimolar) in methanol and acetonitrile. No evidence of photochemical activity was observed after illumination, suggesting the critical role of aggregation in water-rich environments in the onset of such reactions.

Methods

NMR spectroscopy. NMR spectra were acquired on a Bruker Avance DRX 500 MHz spectrometer operating at 500 MHz (^{1}H) and 125 MHz (^{13}C). For each sample, a series of 1D (^{1}H, ^{13}C, and ^{19}F) and 2D (^{1}H–^{1}H and ^{1}H–^{13}C correlation) spectra were obtained in deuterated chloroform. Chemical shifts are given in ppm relative to the solvent peak CDCl3 (^{1}H: δ = 7.26 ppm, ^{13}C: δ = 77.0 ppm). The following abbreviations were used to explain the multiplicities: s = singlet, d = doublet, t = triplet, q = quintet, m = multiplet.

High Resolution Mass Spectrometric (HRMS) analysis. Individual solutions of 2 mg L^{-1} were prepared in methanol LC MS grade and infused directly into a hybrid Quadrupole Time-of-Flight (QToF) mass spectrometer (Maxis Impact, Bruker Daltonics, Bremen, Germany), under a constant flow of 180 μL min^{-1}. The QToF system was equipped with an electrospray ionization interface (ESI), operating in positive ionization mode, with the following operation parameters: capillary voltage 2500 V; end plate offset −500 V; nebulizer pressure 1.0 bar; drying gas 4.0 L min−1; and gas temperature 180 °C. Full scan acquisition mode with a scan rate at 1 Hz was employed, recording spectra over the m/z range 50-1000. A regular system maintenance protocol was followed to assure the good operation of the HRMS system, before analysis, including a QToF external calibration using a sodium formate solution (10 mM), producing Na(NaCOOH)1−14 clusters over the analysis m/z range. A control sample (methanol solution) was also infused for subtracting any background noise from the acquired spectra. The identification relied on the mass accuracy of the precursor ion (<3 mDa) and the isotopic fitting to the theoretical profile (expressed as mSigma value; the lower the value, the better the fitting: <50 excellent fitting, <100 good fitting.

Electron Paramagnetic Resonance (EPR) Experiments. *Chemicals*: The spin trap DMPO (5,5-dimethyl-1-pyrroline N-oxide) was purchased from Sigma Aldrich. EPR measurements were obtained with a Bruker ESP 380E spectrometer equipped with a rectangular ER 4102ST cavity. The microwave frequency was measured with a HP 5350B microwave frequency counter. Samples were placed in capillary glass tubes of internal diameter 0.9 mm and spectra were collected at room temperature and with the following conditions: microwave frequency,

9.773 GHz; microwave power, 20.9 mW; modulation frequency, 100 kHz; modulation amplitude 0.1 mT; centre field at 348.5 mT with a sweep range of 10 mT, and a total 4.9 min scan time over 14 accumulations. EPR spectra were analyzed and simulated using the EasySpin package.[6] Hyperfine coupling constants for aqueous reactive oxygen adducts with DMPO were taken from the literature.

Cyclic Voltammetry. The cyclic voltammetry was conducted on a Versa STAT4 potentiostatgalvanostat with platinum (Pt) disk, Pt wire, and Ag/AgNO3 electrode as the working electrode, counter electrode, and reference electrode, respectively using a 0.1 M solution of tetrabutylammonium hexafluorophosphate (n-Bu4NPF$_6$) in anhydrous dichloromethane or a 0.1 M solution of potassium nitrate (KNO3) in the acetonitrile/water mixtures at a potential scan rate of 20 mV s-1. Films of aggregates were prepared on ITO by drop casting 100 μL of aggregates solution (50 μM; f_w = 98%) on a 0.5 cm^2 area of ITO following by drying in the dark for ~ 6 h. Prior to each experiment, the cell was purged by Ar for at least 5 min. All experiments were conducted under flowing argon.

X-ray Crystallography. Datasets from several crystals of the dimer **2-OH**, crystallised by slow evaporation of a solution of **2-OH** in hexane/dichloromethane, were collected at 100°K, at Beamline P13 of the EMBL-Hamburg synchrotron radiation source. The best-diffracting crystal from hexane was of approximate size 200x80x40 μm and diffracted up to a resolution of 0.77 Å. The polar solvent yielded much smaller crystals, of markedly worse quality. The best-diffracting one was of approximate size 80x40x30 μm and diffracted up to 0.94 Å. Data were processed with the XDS software.[7] Both crystals belonged to space group P2$_1$/c with quasi-identical unit cell dimensions of a=8.76, b=17.41, c=10.66, β=92.15° and a=8.80, b=17.40, c=10.70, β=92.4° for the crystals grown from apolar and polar solvents, respectively. The structures were solved by direct methods with SHELXS and refined with SHELXL.[8] Refinement converged to R-factors R1=6.60% and R1=9.85% (for reflections with Fo>4σ(Fo)) for hexane and the polar solvent, respectively. All non-hydrogen atoms were modeled from experimental peaks. Hydrogens were modeled at calculated positions, except for the two hydrogens of the CH-OH group that were not included in either structure. In both structures, the asymmetric unit consists of half the dimer, with the –OH group (only the O was modeled) distributed amongst the two equivalent positions of the two crystallographically symmetric linker carbons.

The X-ray diffraction analysis of microcrystals of BODIPY **1** collected by ultracentrifugation was conducted using a Rigaku SmartLab Diffractometer with Cu-Ka radiation. Θ/2Θ scans were employed and the angular range for data collection was 2.0-60.0°, scanned in steps of 0.02° with a scan speed of 10sec/step.

Steady-State and ns Time-Resolved Fluorescence Spectroscopy. All steady-state absorption measurements were collected using a Perkin-Elmer Lambda-16 and a Cary 50 UV-Vis spectrophotometer. The fluorescence and fluorescence-excitation spectra were recorded using a Perkin-Elmer model LS-50B and a FluoroMax-4 (Horiba Scientific) fluorometer. The latter was used for fluorescence lifetime measurements in a time-resolved configuration. All emission spectra were corrected for the wavelength-dependence sensitivity of the detector. Fluorescence quantum yield measurements were obtained relative to Rhodamine 6G in ethanol (Φ=0.94)[9] and to cyanine dye **cy681** in methanol (Φ=0.24)[10] for the 680-820 nm emission range.

Transient Absorption Setup. For the fs-TA measurements, we employed an amplified Ti:Sapphire laser (Libra Coherent, 800 nm, 80 fs pulse duration, 1 kHz repetition frequency). The details of the experimental apparatus system have been extensively described in ref [11]. A fraction of the fundamental beam was frequency doubled to drive a non-colinear optical parametric amplifier (NOPA), generating approximately 100-fs laser pump pulses centered at 500 nm. The probe pulses spanned from 450 nm to 700 nm and were created by focusing the 800 nm beam onto a thin sapphire plate. Concentrations used matched those in steady-state measurements. Monomeric **BODIPY** solutions (50 μM; f_w = 0% - 80%) and microcrystalline state BODIPY solutions (50 μM; f_w = 95%) were placed in 0.2 mm and 1 mm cuvettes, respectively, yielding < 0.1 OD absorbance at 500 nm. To minimize exciton-exciton annihilation processes in the microcrystalline state, fluence was maintained below 50 μJ/cm^2. To prevent photochemistry or photodamage during the measurements, the samples were shielded from external light sources and kept flowing using a circulating pump. For the ns-TA measurements, we employed an amplified femtosecond laser (Light Conversion Pharos, 1024 nm, 300 fs pulse duration, 2 kHz repetition frequency). Probe pulses spanning 450 nm to 700 nm were generated on a thin sapphire plate. Synchronized 532 nm pump pulses of ~800 ps were produced by the second harmonic of a Q-switched Nd: YVO4 laser (Innolas Picolo). Excitation fluence was adjusted with a neutral density filter for comparable signal amplitudes to fs-TA at nanosecond timescales. All TA data were collected with a magic angle configuration (54.7°) for pump and probe polarizations. For the fitting a global analysis algorithm (Glotaran) was utilized.[12]

Atomic Force Microscopy (AFM). 2x2 μm^2 AFM topographic images of crystalline aggregates were obtained by spin casting a solution of **2-OH** (10 μM; f_w = 95%) onto a quartz plate. The AFM measurements were performed using an NT-MDT AFM system in tapping operation mode.

Scanning Electron Microscopy (SEM). SEM photographs were recorded with a JEOL 7401f 614 FESEM.

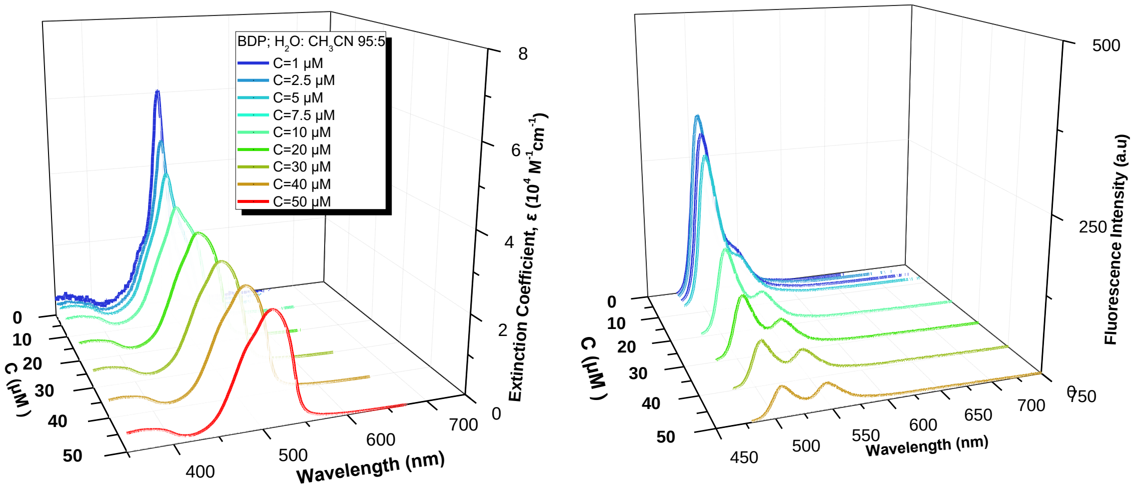

Figure S1. Concentration-dependent absorption (left) and fluorescence spectra (right) of BODIPY **1** in 95/5 (v/v) H2O/MeCN.

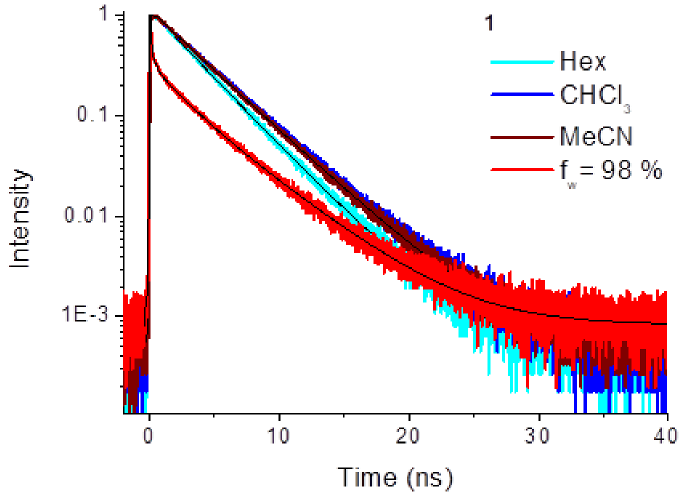

Solvent	A1	τ_1 (ns)	A2	τ_2 (ns)	A3	τ_3 (ns)	τ_{avg} (ns)
MeCN					1.00	3.54	3.54
CHCl3					1.00	3.74	3.74
Hexane					1.00	3.09	3.09
H2O/MeCN (f_w = 98%)	0.72	0.1	0.14	1.1	0.14	4.2	0.8

Figure S2. Fluorescence decay and fitting curves of free BODIPY **1** in different solvents and in its aggregated state (98/2 (v/v) H2O/MeCN), detected at the peaks of the fluorescence spectra. The fluorescence intensity of aggregates at 545 nm decays rapidly with an average lifetime of 0.8 ns (3.54 ns in MeCN;), contributed by three lifetimes of 0.1 ns (72%), 1.1 ns (14%), and 4.2 ns (14%).

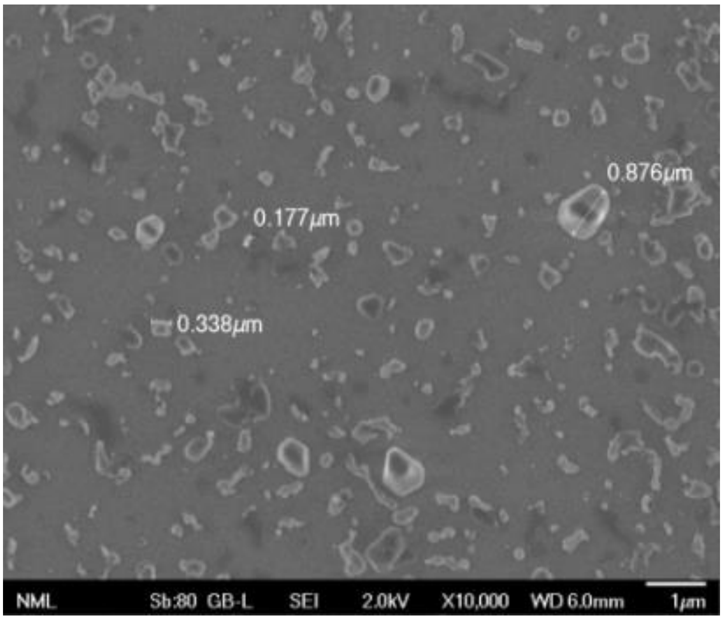

Figure S3. SEM photograph obtained from a 50 μM solution (fw=98%) of BODIPY **1** spun onto a quartz plate (3000 rpm).

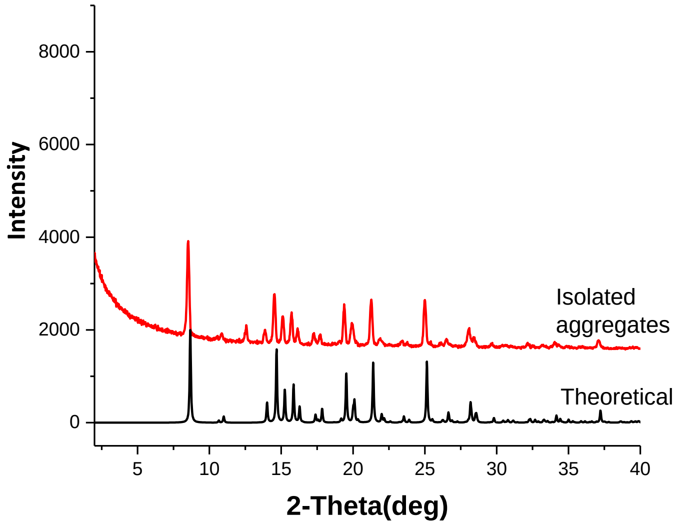

Figure S4. BODIPY **1** at *rt* crystallizes in the orthorhombic Pbca space group, and the theoretically calculated pattern using Mercury, is presented in black. The experimentally measured pattern from the crystalline aggregates isolated by ultracentrifugation (40000 G) from an aqueous solution is shown in red.

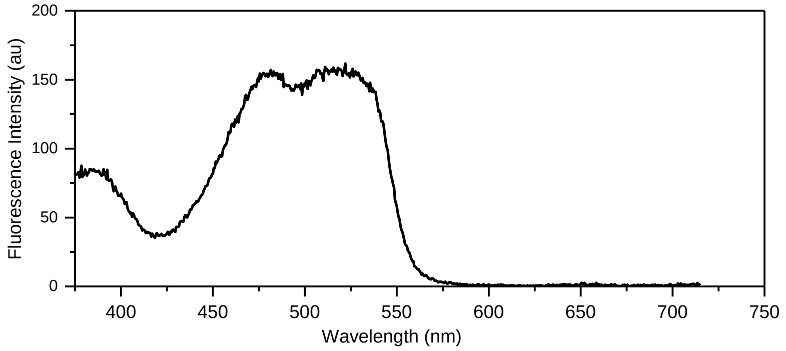

Figure S5. Fluorescence - excitation spectrum monitored at 721 nm upon illuminating a 50.0 μM aqueous solution (3.0 mL; f_w = 98%) of BODIPY **1**.

Characterization of photoproducts: NMR spectra and Mass Spectrometry

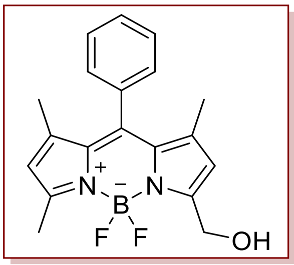

1-OH Yield: 3,8 mg, 5.6 %.

^{1}H NMR (500 MHz, $CDCl_3$): δ = 7.50 (m, 3H; m, p), 7.29 (m, 2H; o), 6.20 (s, 1H; H5), 6.05 (s, 1H; H2), 6.81 (d, J = 5 Hz, 2H; H6), 2.68 (m, 1H; H7), 2.57 (s, 3H; H1), 1.41 (s, 6H; H4, H3).

^{13}C NMR (125 MHz, $CDCl_3$): δ = 145.6 (C), 143.6 (C), 129.3 - 127.7 (ArC), 122.4 (C2), 119.5 (C5). 57.9 (C6), 14.8 (C1), 14.6 (C4), 14.3 (C3).

^{19}F NMR (376 MHz, $CDCl_3$): δ = -143.81 (q, J_{BF} = 33 Hz, 2F; 1 x BF_2).

HRMS: $[M+Na]^+$ was identified (theoretical m/z: 363.1451) with a mass error of 0.6 ppm and excellent isotopic fitting (mSigma: 21).

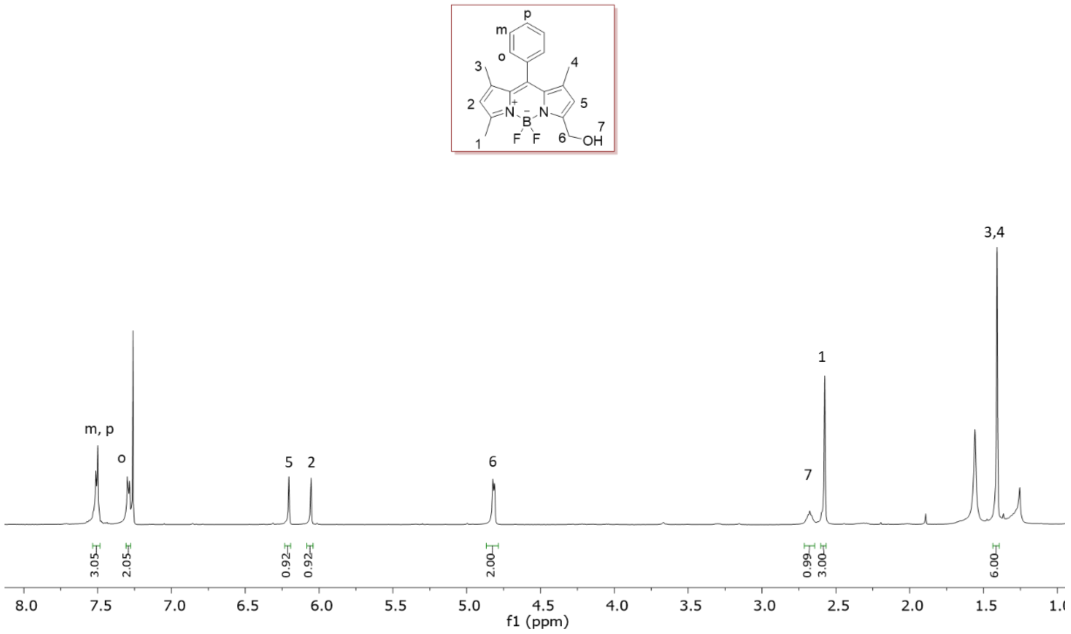

Figure S6. ^{1}H-NMR (500 MHz, $CDCl_3$) spectrum of **1-OH**.

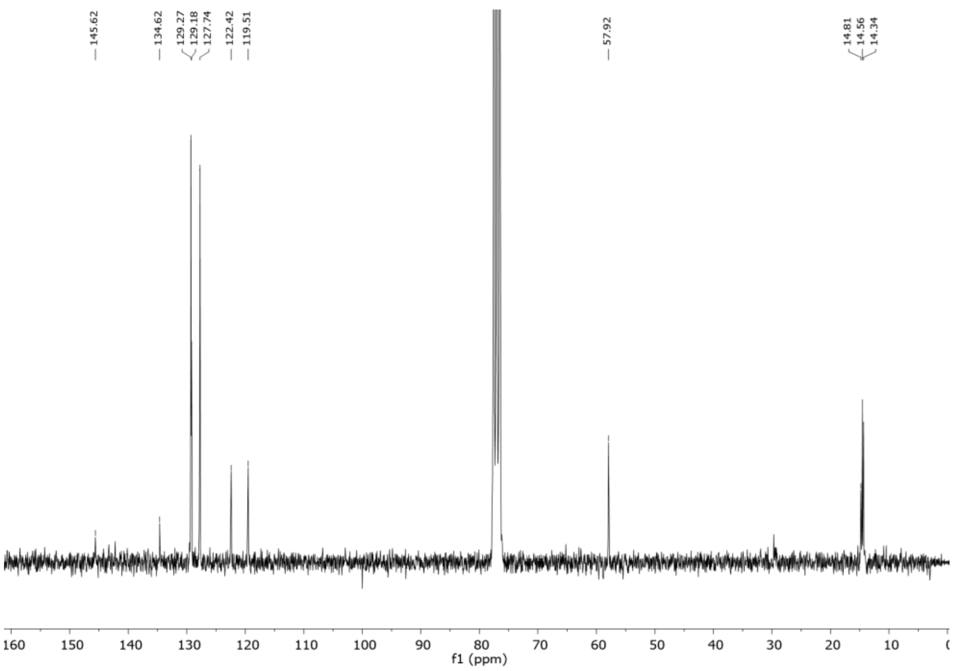

Figure S7. ^{13}C-NMR (125 MHz, $CDCl_3$) spectrum of **1-OH**.

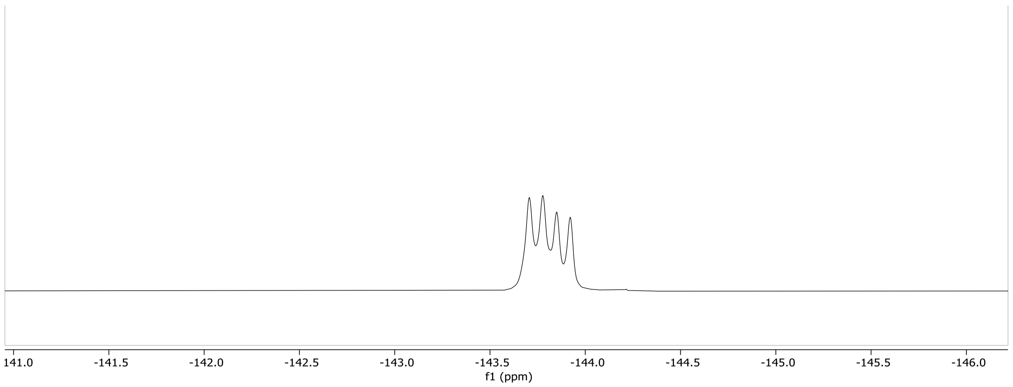

Figure S8. ^{19}F-NMR (376 MHz, $CDCl_3$) spectrum of **1-OH**.

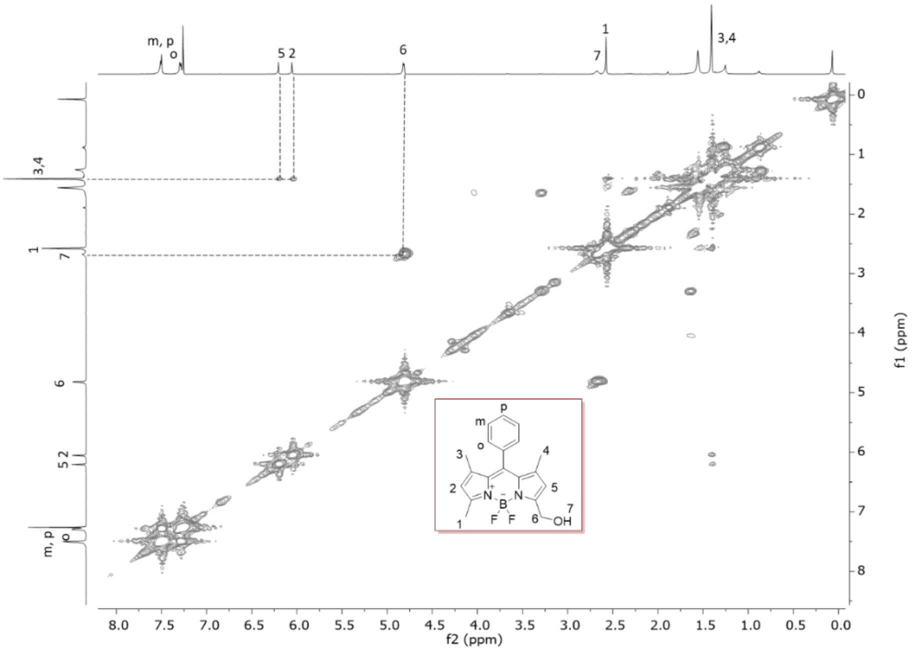

Figure S9. ^{1}H-^{1}H-COSY NMR (500 MHz, $CDCl_3$) spectrum of **1-OH**.

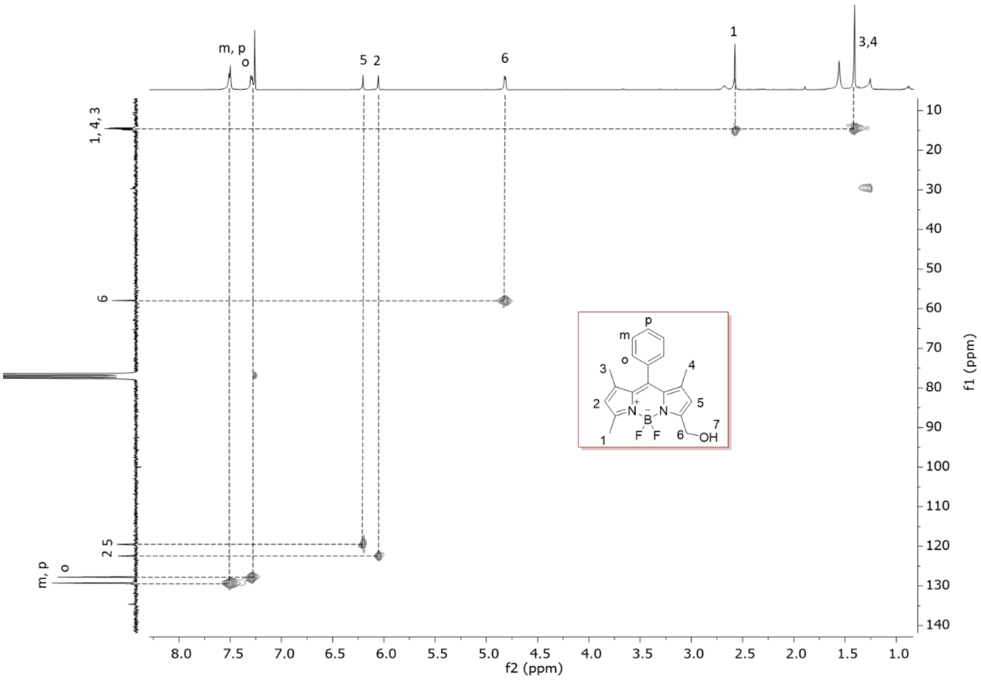

Figure S10. ^{1}H-^{13}C HSQC NMR spectrum of **1-OH**.

HRMS

1-OH; [M+Na]$^+$ = 363.1451

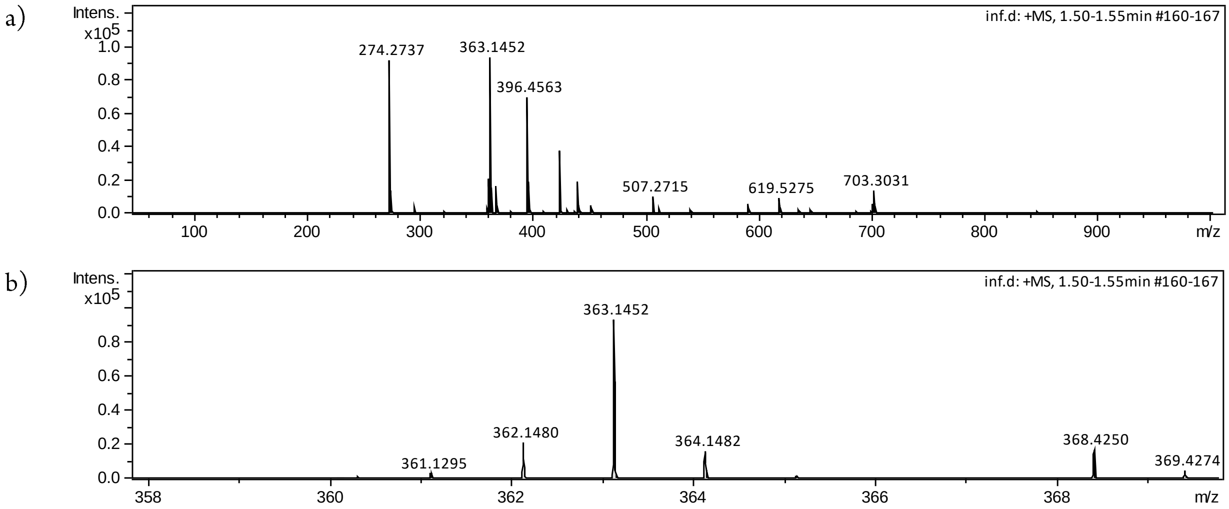

Figure S11. HRMS spectra of **1-OH** (**a**) m/z range 50-1000 and (**b**) zoomed in the range of the precursor ion.

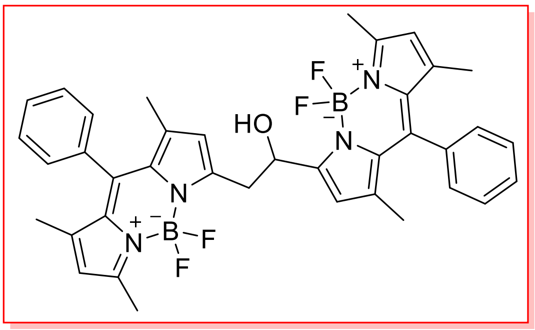

2-OH Yield: 10,9 mg, 8.2 %.

^{1}H NMR (500 MHz, $CDCl_3$): δ = 7.49 (m, 6H; m, p), 7.30 (m, 2H; o), 6.40 (s, 1H; H5), 6.32 (s, 1H; H9), 6.03 (s, 1H; H2/H12), 5.99 (s, 1H; H2/H12), 5.59 (m, 1H; H7), 3.68 (dd, J = 5 Hz, J = 16 Hz, 1H; H6a), 3.52 (dd, J = 9 Hz, J = 16 Hz, 1H; H6b), 3.43 (m, 1H; H8), 2.58 (s, 3H; H1), 2.56 (s, 3H; H13), 1.39 (m, 12H; H4, H10, H11, H3).

^{13}C NMR (125 MHz, $CDCl_3$): δ = 157.9 (C), 157.8 (C), 156.0 (C), 155.3 (C), 145.1 (C), 143.7 (C), 143.3 (C), 142.6 (C), 135.0 (C), 134.8 (C), 129.2 - 127.8 (ArC), 122.2 (C2), 121.4 (C12), 120.6 (C5), 117.7 (C9), 66.2 (C7), 34.7 (C6), 15.0 – 14.4 (C1, C13, C3, C4, C10, C11).

^{19}F NMR (376 MHz, $CDCl_3$): δ = -143.34 (dq, J_{BF} = 33 Hz, $J_{F\text{-}F}$ = 104 Hz, F; BF_2), -142.24 (q, J_{BF} = 33 Hz, 2F; 1xBF_2), -141.34 (dq, J_{BF} = 33 Hz, $J_{F\text{-}F}$ = 104 Hz, F; 1xBF_2).

HRMS: $[M+Na]^+$ was identified (theoretical m/z: 685.2916) with a mass error of -0.1 ppm and excellent isotopic fitting (mSigma: 50).

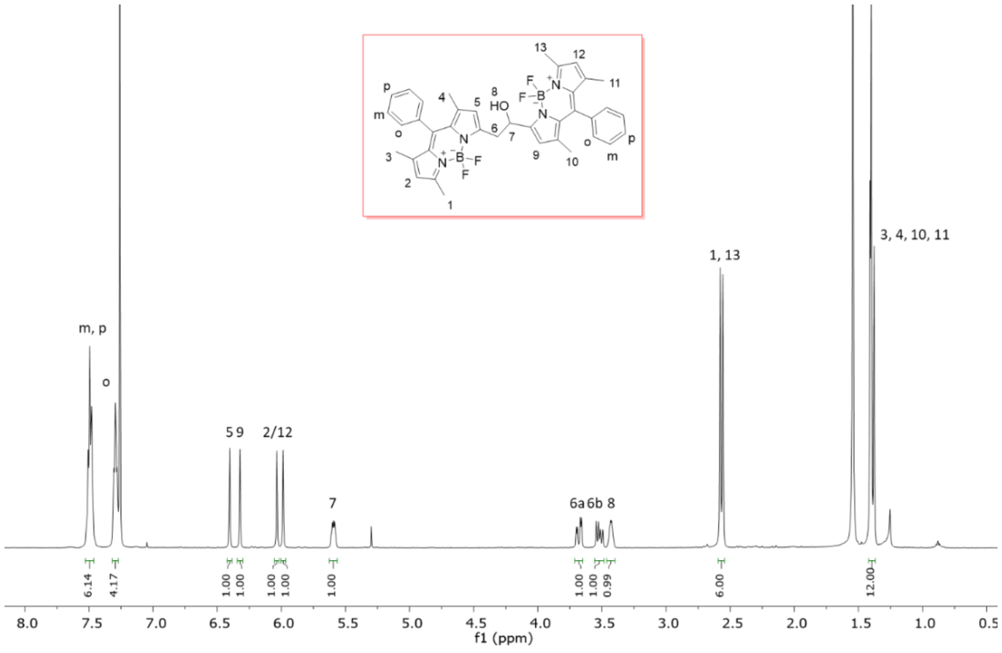

Figure S12. ^{1}H-NMR (500 MHz, $CDCl_3$) spectrum of **2-OH**.

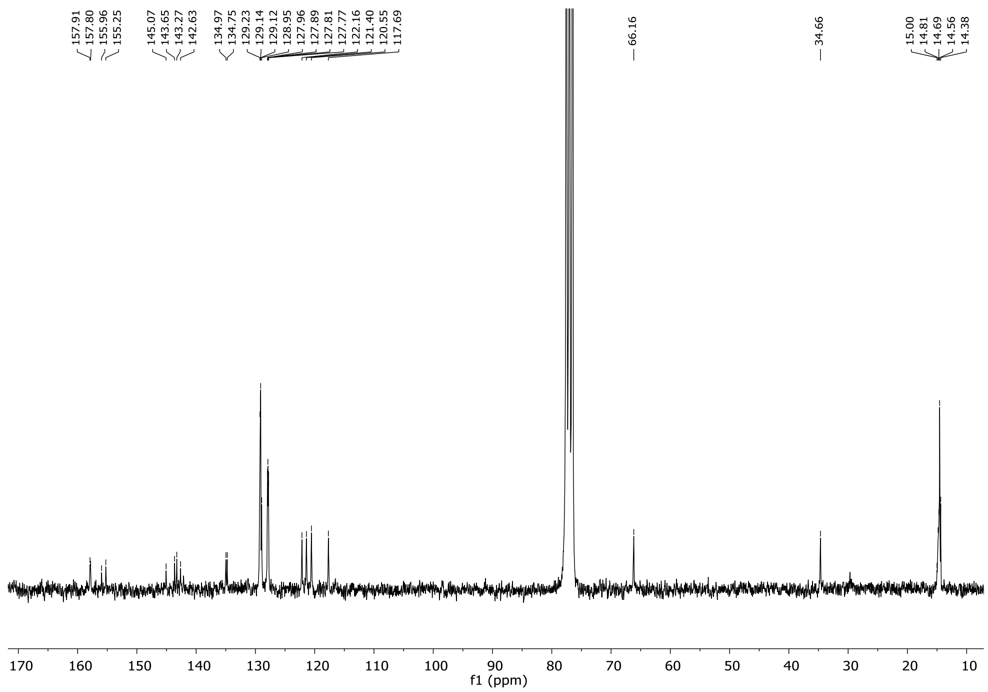

Figure S13. ^{13}C-NMR (125 MHz, $CDCl_3$) spectrum of **2-OH**.

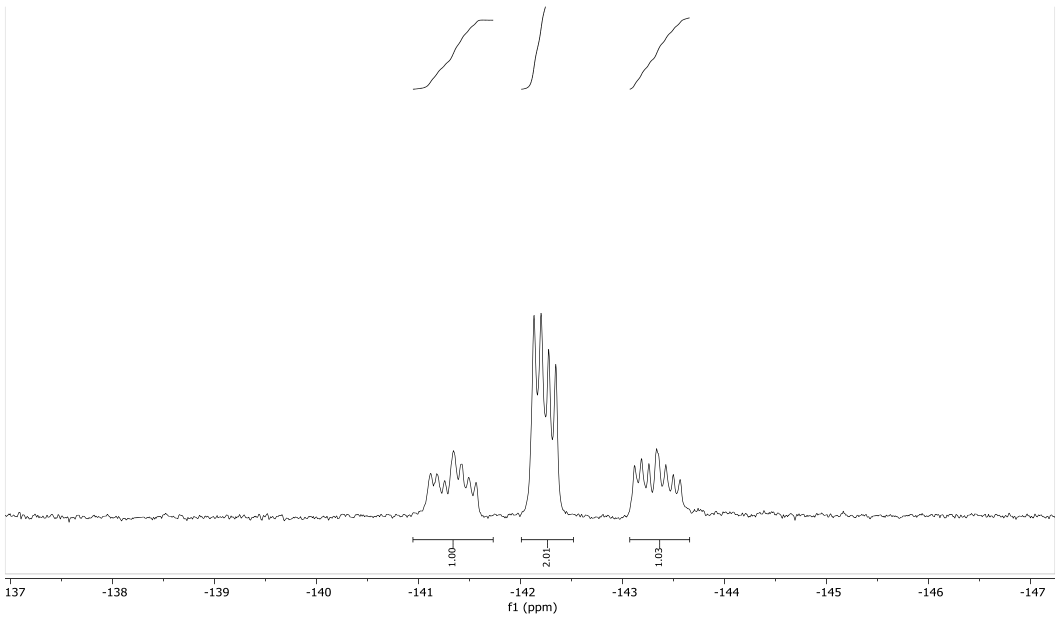

Figure S14. ^{19}F-NMR (376 MHz, $CDCl_3$) spectrum of **2-OH**.

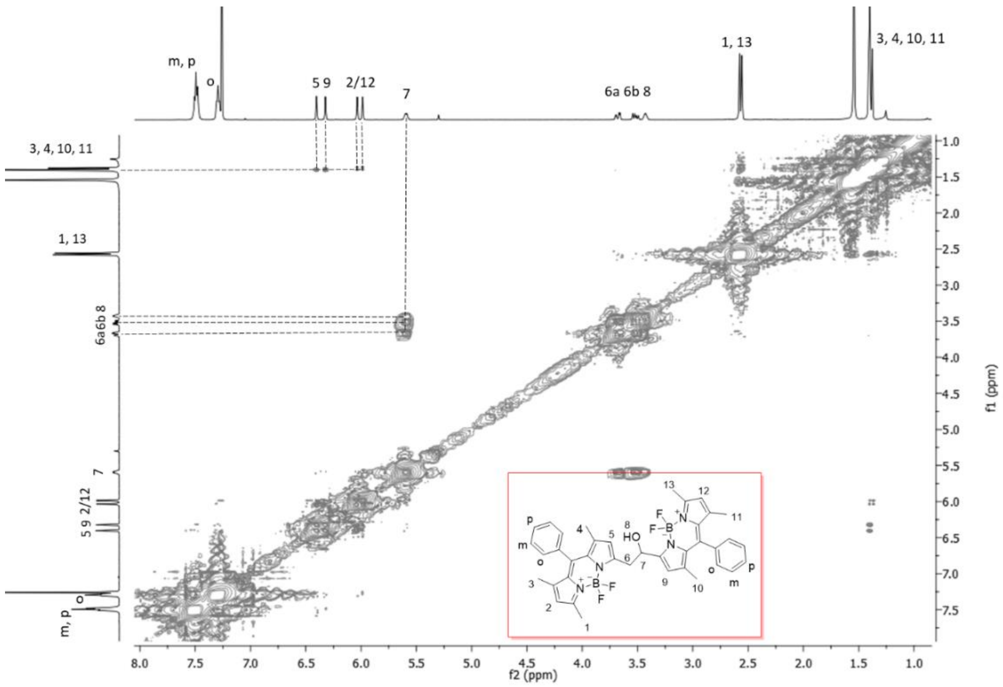

Figure S15. ^{1}H-^{1}H COSY NMR (500 MHz, $CDCl_3$) spectrum of **2-OH**.

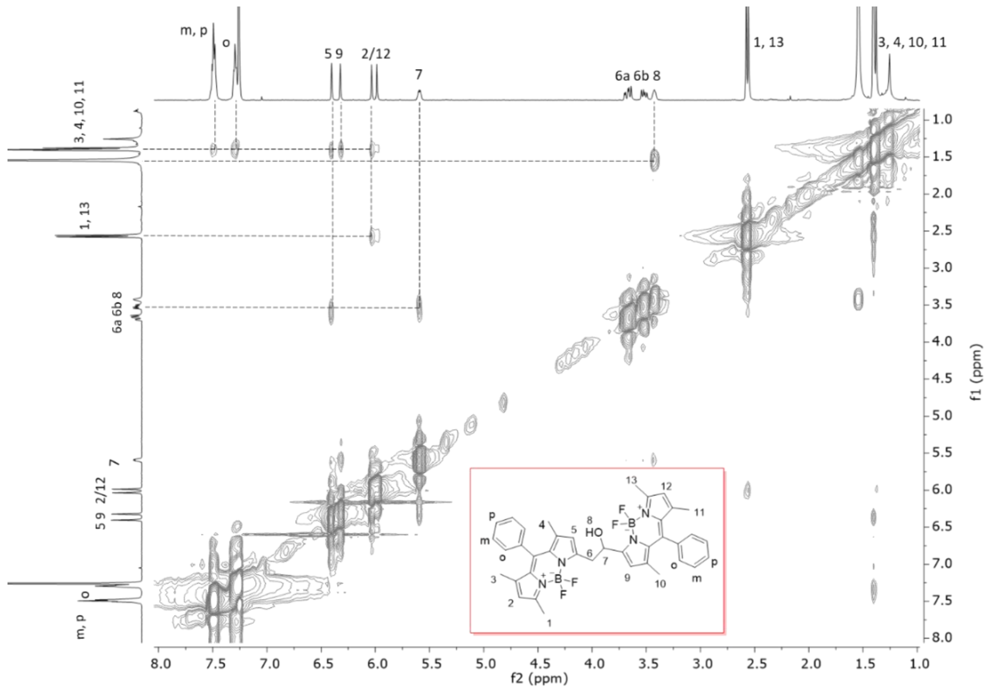

Figure S16. ^{1}H-^{1}H ROESY NMR (500 MHz, $CDCl_3$) spectrum of **2-OH**.

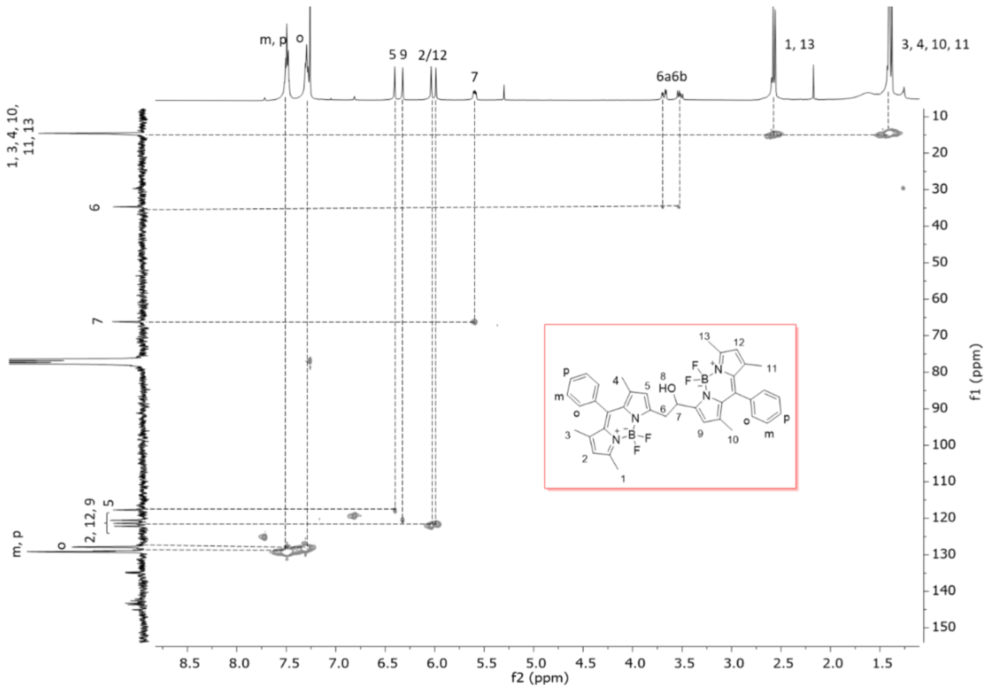

Figure S17. ^1H-^{13}C HSQC NMR spectrum of **2-OH**.

HRMS

2-OH; $[M+Na]^+$ = 685.2916

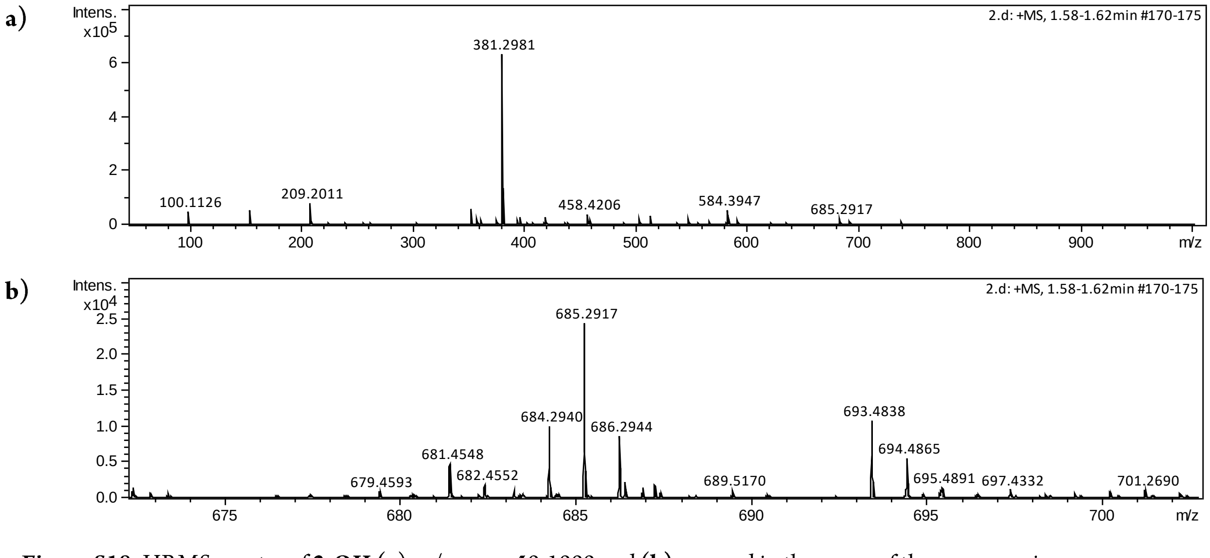

Figure S18. HRMS spectra of **2-OH** (**a**) m/z range 50-1000 and (**b**) zoomed in the range of the precursor ion.

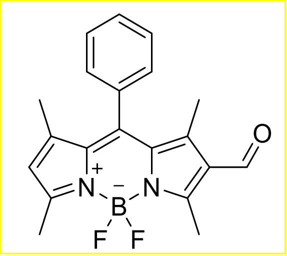

bp1 Yield: 0,7 mg, ~1 %.

^{1}H NMR (500 MHz, $CDCl_3$): δ = 10.01 (s, 1H; C5), 7.56-7.50 (m, 3H; m, p), 7.32-7.25 (m, 2H; o), 6.15 (s, 1H; H2), 2.82 (s, 3H; CH3), 2.62 (s, 3H; CH3), 1.65 (s, 3H; CH3), 1.42 (s, 3H; CH3).

^{13}C NMR (125 MHz, $CDCl_3$): δ = 185.9 (C9), 161.6 (C), 156.5 (C), 147.3 (C), 143.6 (C), 142.9 (C), 134.2 (C), 129.5 (o, p), 127.7 (m), 126.3, 124.0 (C2), 15.1 (CH3), 14.8 (CH3), 13.0 (CH3), 11.6 (CH3).

^{19}F NMR (376 MHz, $CDCl_3$): δ = 144.76 (q, J_{BF} = 32 Hz, 2F; 1 x BF_2).

HRMS: $[M+H]^+$ was identified (theoretical m/z: 353.1635) with a mass error of -0.1 ppm and excellent isotopic fitting (mSigma: 41).

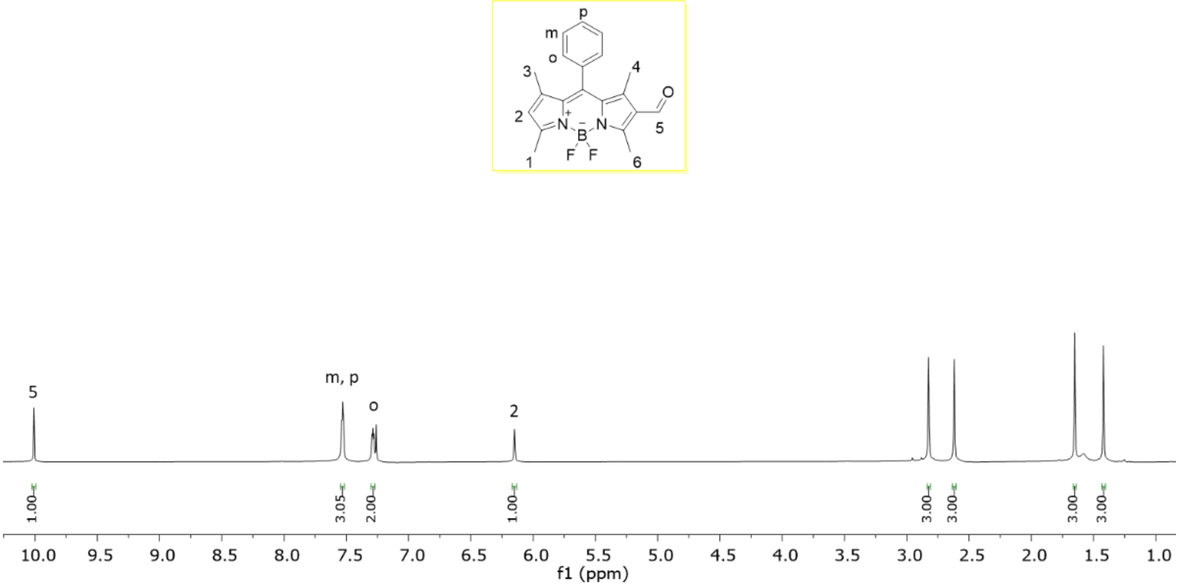

Figure S19. ^{1}H-NMR (500 MHz, $CDCl_3$) spectrum of b**p1**.

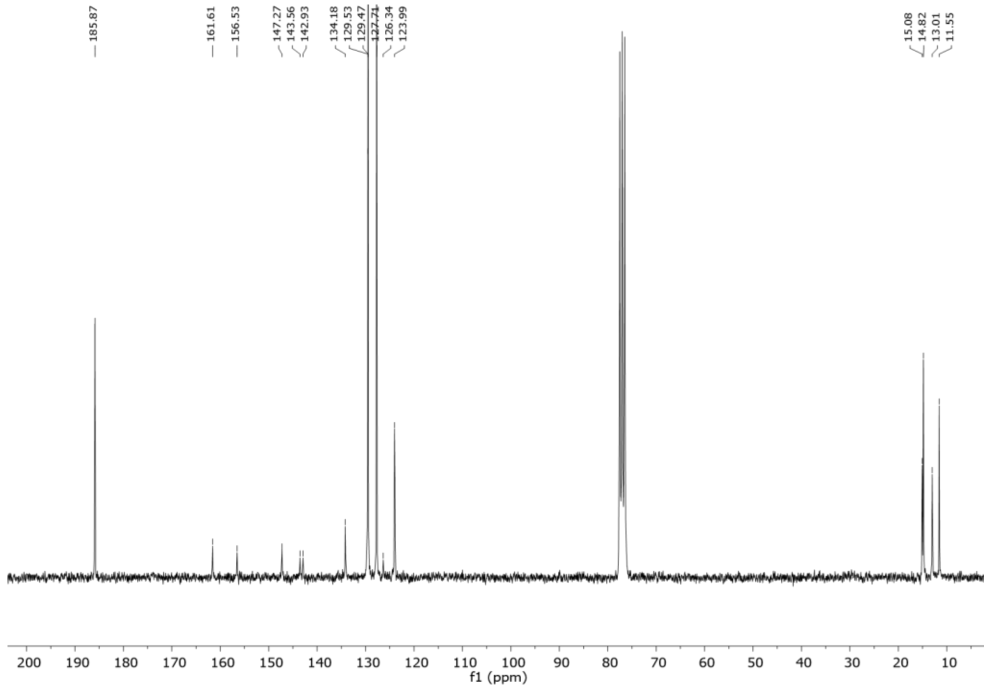

Figure S20. ^{13}C-NMR (125 MHz, $CDCl_3$) spectrum of b**p1**.

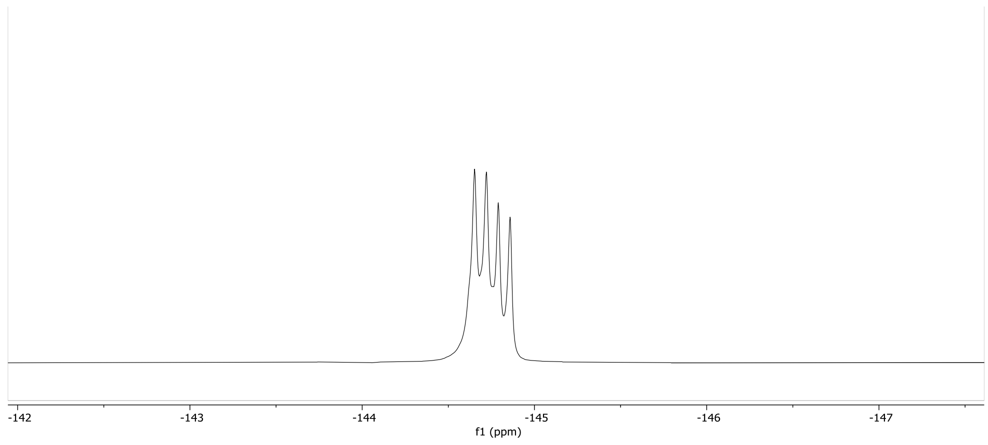

Figure S21. ^{19}F-NMR (376 MHz, $CDCl_3$) spectrum of **bp1**.

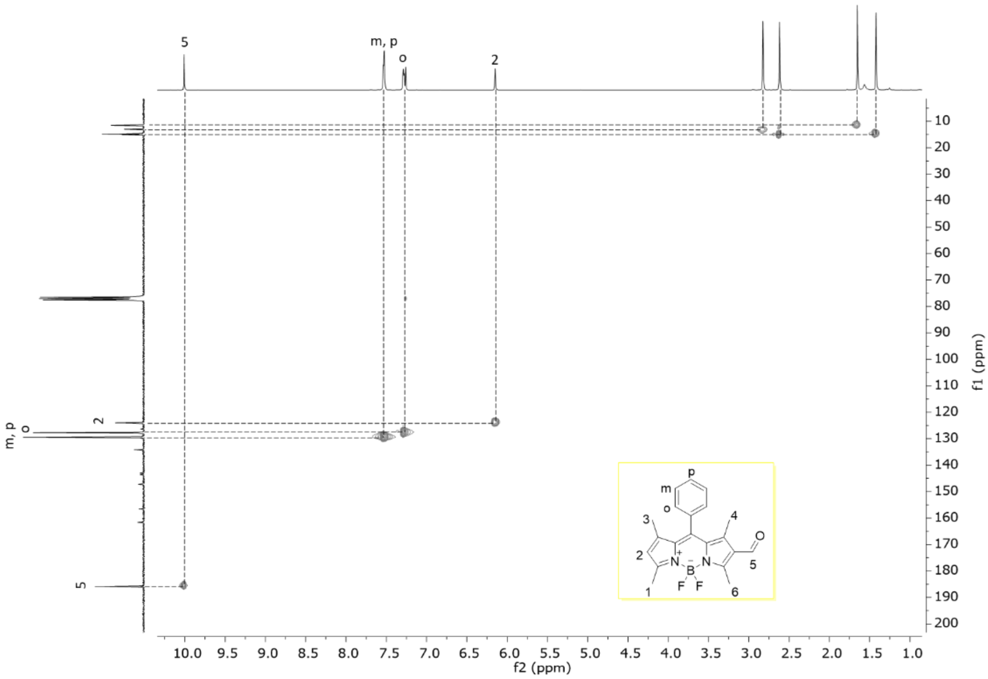

Figure S22. ^{1}H -^{13}C HSQC NMR spectrum of b**p1**.

HRMS

Bp1; [M+H]$^{+}$ = 353.1635

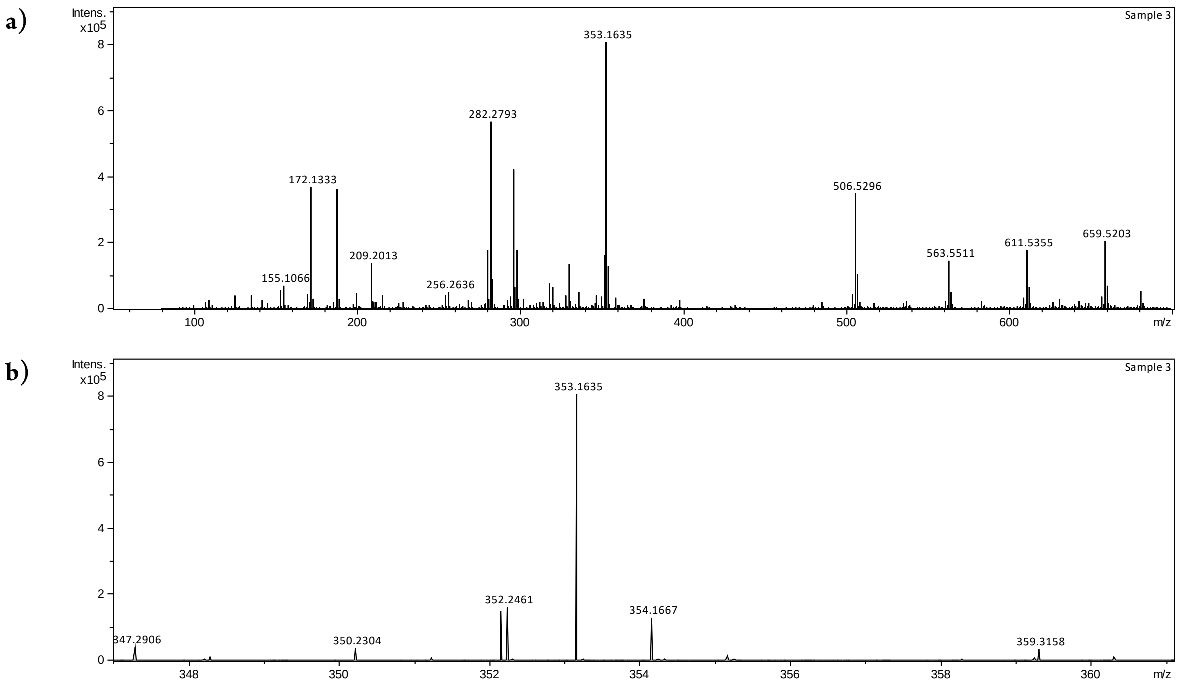

Figure S23. HRMS spectra of **bp1** (**a**) m/z range 50-1000 and (**b**) zoomed in the range of the precursor ion.

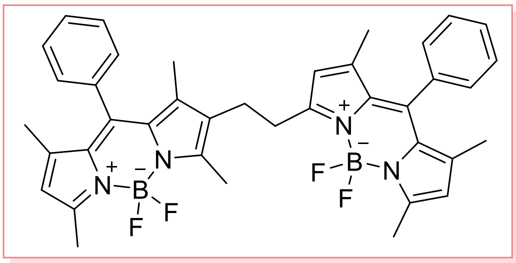

bp2: (thermally unstable to elevated temperatures) Yield: 2 mg, 1.5 %.

^{1}H NMR (500 MHz, $CDCl_3$): δ = 7.49 (m, 6H; m, p), 7.29 (m, 4H; o), 6.00 (s, 1H; H2), 5.97 (s, 1H; H11), 5.61 (s, 1H; H8), 4.04 (s, 2H; H7), 2.57 (s, 3H; H1), 2.56 (s, 3H; H12), 2.53 (s, 3H; H5), 1.38 (s, 3H; H3), 1.37 (s, 3H; H10), 1.32 (s, 3H; H4), 1.31 (s, 3H; H9), 1.25 (br, 2H; H6).

^{13}C NMR (125 MHz, $CDCl_3$): δ = 156.2-134.8 (C), 129.1-127.9 (ArC), 121.6 (C2), 121.1 (C11), 119.5 (C8), 29.7 (C6), 23.2 (C7), 14.6 (C12), 14.4 (C1, C3, C10, C9), 12.7 (C5), 12.0 (C4).

^{19}F NMR (376 MHz, $CDCl_3$): δ = -145.46 (q, J_{BF} = 35 Hz, 2F; BF_2), -145.98 (q, J_{BF} = 30 Hz, 2F; BF_2).

HRMS $[M\text{-}CH_2\text{+}Na]^+$ was identified (theoretical m/z: 669.2967) with a mass error of 2.6 ppm and excellent isotopic fitting (mSigma: 41).

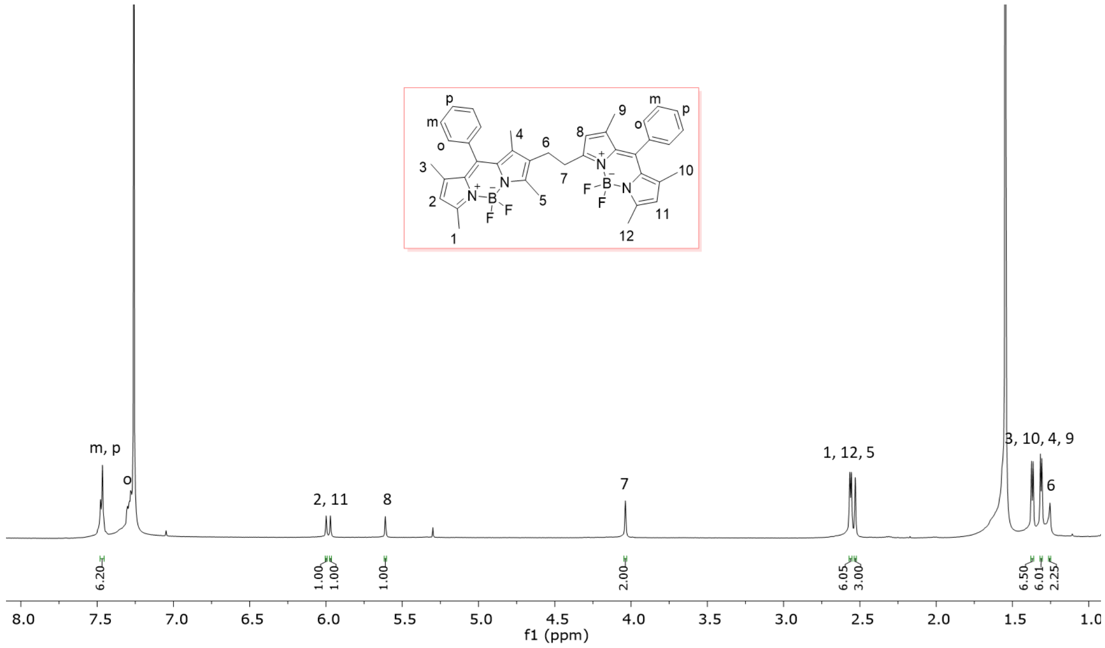

Figure S24. ^{1}H NMR (500 MHz, $CDCl_3$) spectrum of **bp2.**

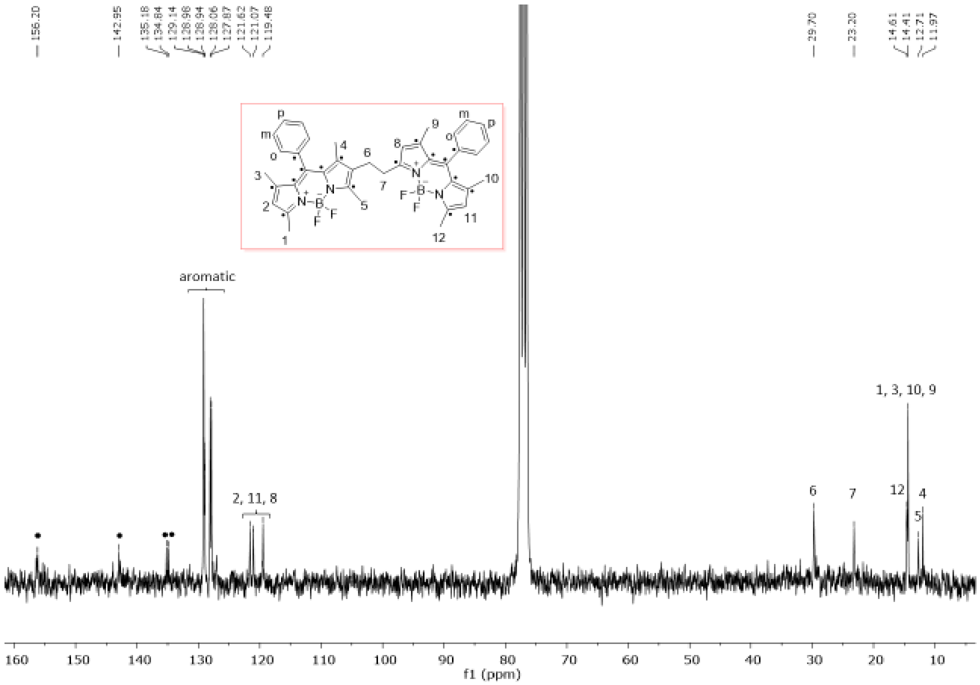

Figure S25. ^{13}C-NMR (125 MHz, $CDCl_3$) spectrum of **bp2**.

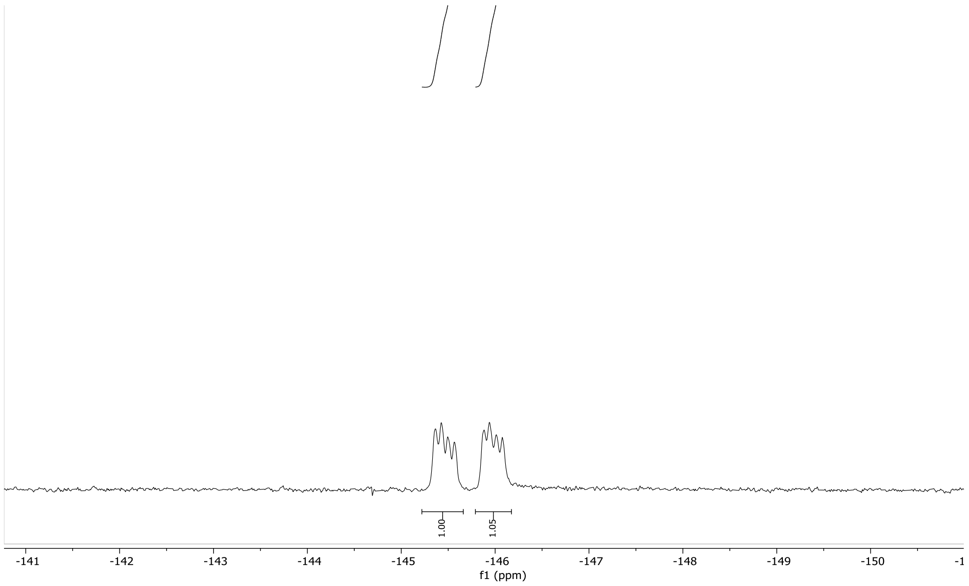

Figure S26. ^{19}F-NMR (376 MHz, $CDCl_3$) spectrum of bp2.

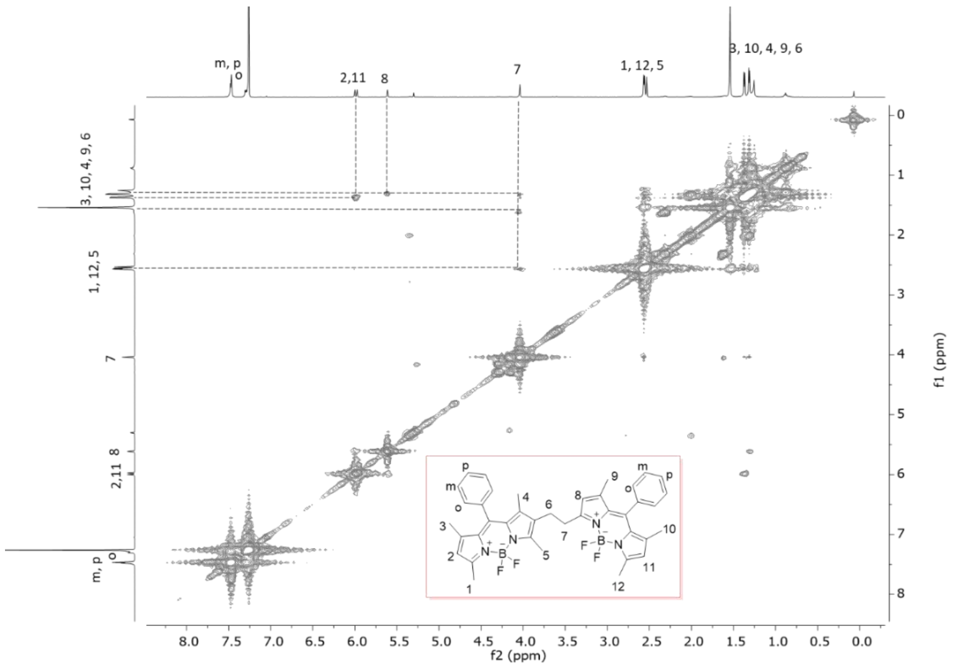

Figure S27. ^{1}H-^{1}H COSY NMR (500 MHz, $CDCl_3$) spectrum of **bp2**.

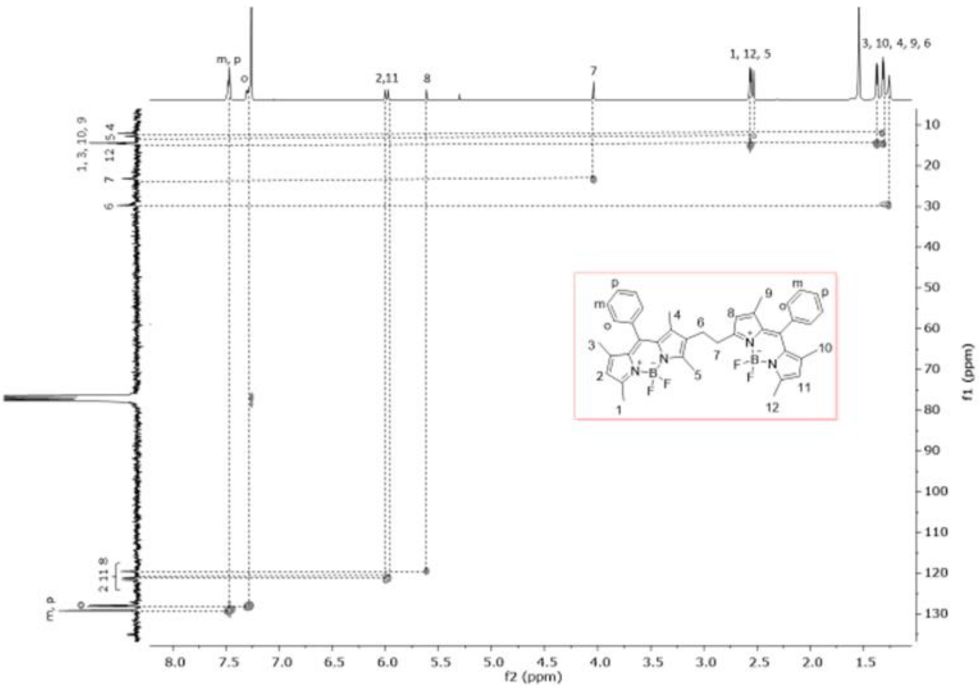

Figure S28. ^{1}H-^{13}C HSQC NMR spectrum of **bp2.**

HRMS

Bp2; $[M\text{-}CH_2+Na]^+$ = 669.2967

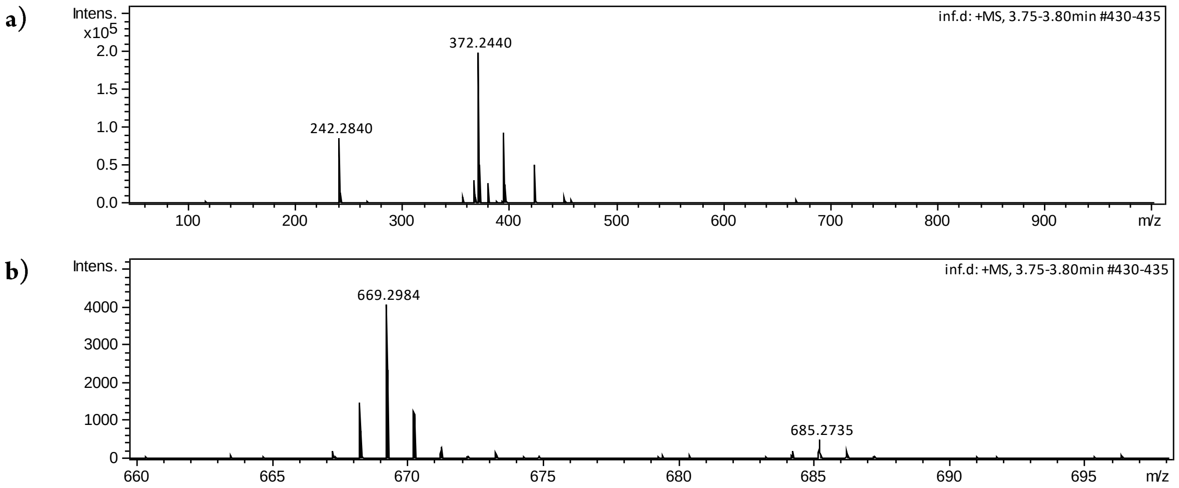

Figure S29. HRMS spectra of **bp2** (**a**) m/z range 50-1000 and (**b**) zoomed in the range of the precursor ion.

Electron Paramagnetic Resonance (EPR)

The degraded DMPO radical shown in *Figure 3c* of the main text, is present along with a new signal denoted by empty rectangles (□). The latter comprises a six-line spectrum which following its spectral fitting can be reproduced assuming two hyperfine coupling constants, namely 15.8 and 22.6 Gauss. By assigning these values to N and H nuclei we may infer that the trapped radical can be a carbon-centered methyl or ethyl species.[13]

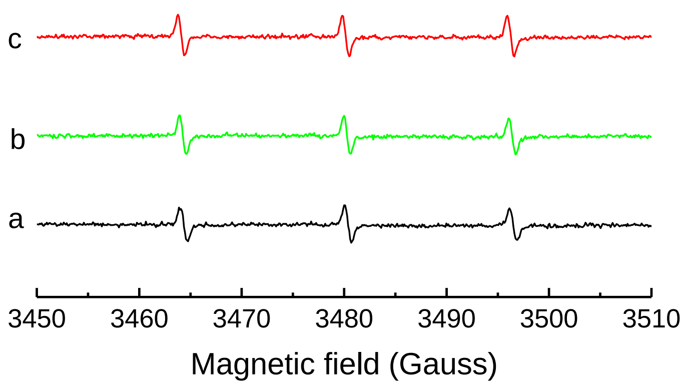

Figure S30. EPR spectra of aggregates (f_w = 98%; [**1**] = 50 μM) in the presence of 100 mM 4-oxo-TEMP. (a) Control sample containing the spin trap in water, (b) sample of aggregates plus spin trap, before illumination, (c) as in (b) following 5 min illumination. It is noted that the three-line signal observed in the control experiment is a trace admixture attributed to Tempone contamination, and probably is due to slow air oxidation of solid 4-oxo-TEMP during storage.

Femtosecond Transient Absorption (Fs-TA) Spectroscopy

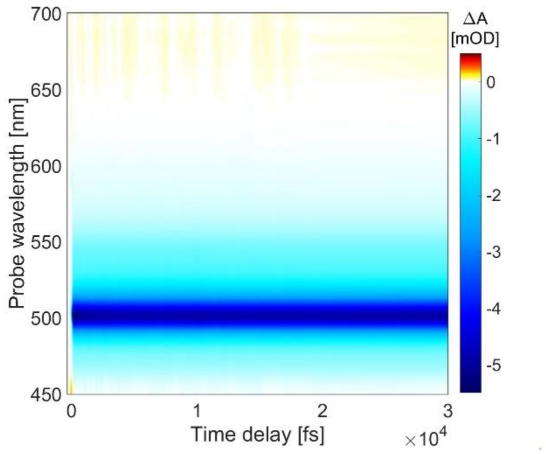

Figure S31. 2D-plots of the TA spectra for **1** in MeCN.

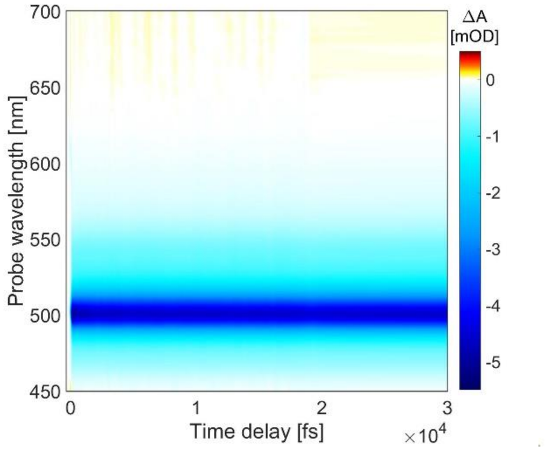

Figure S32. 2D-plots of the TA spectra for **1** in 70% water.

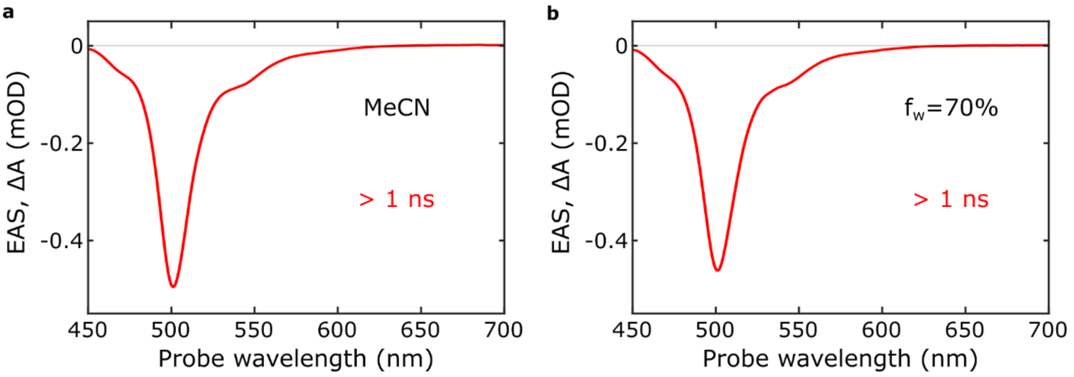

Figure S33. Evolution associated spectra (EAS) and their corresponding time constants, retrieved through global analysis of the TA datasets for **1,** are depicted (a) in MeCN and (b) in 70% water.

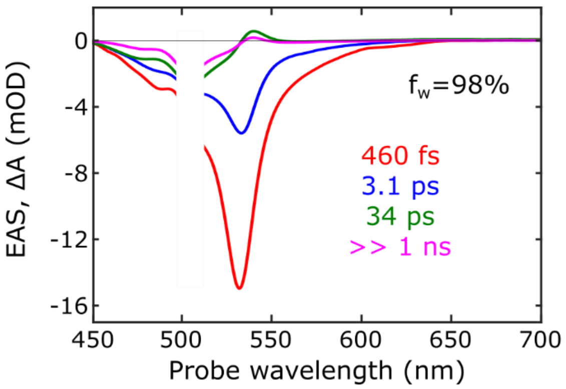

Figure S34. EAS components and their corresponding time constants, retrieved through global analysis of the TA datasets for **1** in 98% water upon 500 nm excitation. The datasets under these conditions are presented in the main text. The probe region exhibiting strong pump scattering around 500 nm, attributed to the presence of aggregates, has been omitted.

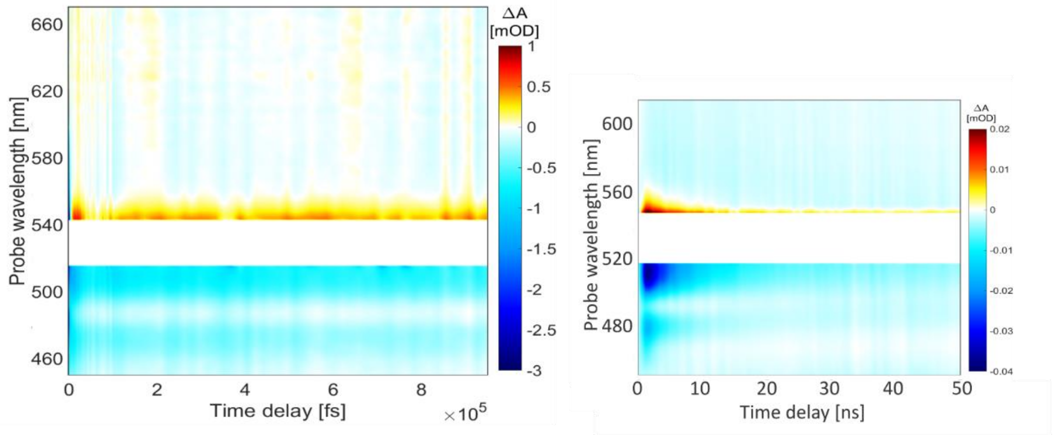

Figure S35. 2D-plots of the TA spectra for **1** in 98% water, spanning from sub-ns (left) to tens of ns timescales (right). The TA maps are acquired with excitations at 530 nm (left) and 532 nm (right), respectively. The probe region, which shows strong pump scattering around 530 nm due to the presence of aggregates, has been omitted.

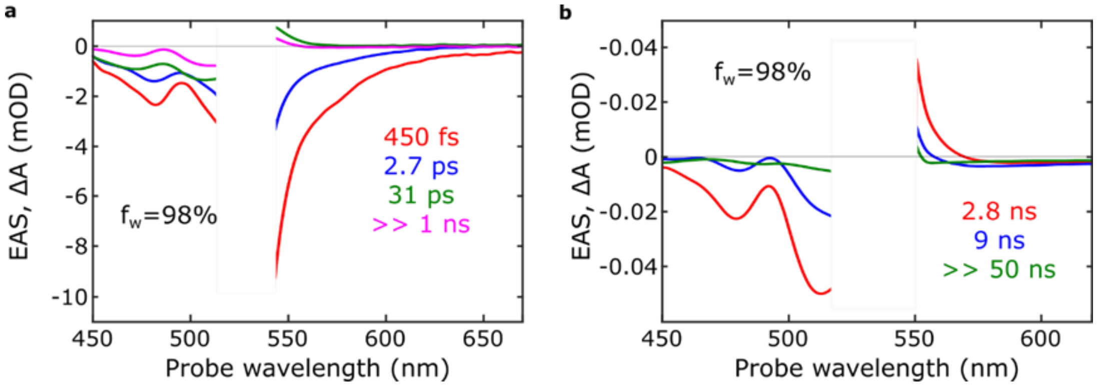

Figure S36. EAS components and their corresponding time constants, retrieved by global analysis of the TA datasets for **1** in 98% water, exploring the (a) sub-ns to (b) tens of ns timescales. The probe region, which shows strong pump scattering around 530 nm due to the presence of aggregates, has been omitted.

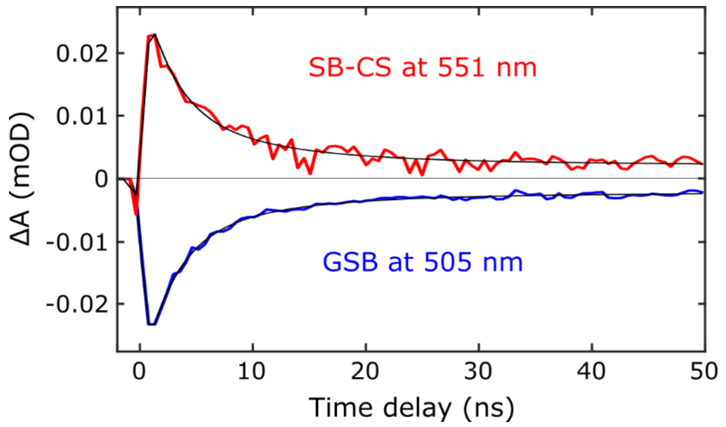

Figure S37. Kinetic traces of the ground state bleaching (GSB) signal at 505 nm (blue) and the symmetry-breaking charge separated (SB-CS) state at 551 nm (red) overlaid with their fits (black solid lines), extracted by the global analysis shown in Figure S36b.

Table S1. Decay time constants of the SB-CS of **1** in 98% of water, combining the timescales identified in the global analysis presented in Figure S36.

**BODIPY 1**	**A_1 (%)**	**τ_1 (ps)**	**A_2 (%)**	**τ_2 (ns)**	**A_3 (%)**	**τ_3 (ns)**	**A_4 (%)**	**τ_4 (ns)**	**$\langle\tau\rangle^a$ (ns)**
**SB-CS decay**	44	31	33	2.8	17	9	6	> > 50	2.6

[a] Taken as the weighted average of the three decay components of the SB-CS. The weights are assigned based on the percentage of the recovery of the integrated GSB response for each process. The aforementioned decay components were determined using apparatus systems with sub-ns (for τ_1) and tens of ns (for τ_2 and τ_3) time delays. The long-living non-decaying component, appearing as a plateau in our timescales, was not included in these calculations**.**

Table S2. Comparison of the charge separation and charge recombination (CR) rates of the SB-CS state observed for **1** in 98% of water.

**BODIPY 1**	**$\tau_{SB\text{-}CS}$ (ps)**	**$\langle\tau_{CR}\rangle^a$ (ns)**	**$k_{SB\text{-}CS}/k_{CR}$**
**SB-CS**	3	> 5.4	> 1800

[a] Taken as the weighted average of all the components identified in the global analysis, namely: 31 ps, 2.8 ns, 9 ns and the component exceeding 50 ns.

As mentioned in the main text, the manifestation of the symmetry-broken charge separated (SB-CS) state in BODIPY pairs is characterized by the presence of the excited state absorption (ESA) band centered around 540 nm.[14] The quenching of the stimulated emission (SE) of the BODIPY **1** aggregates in 98% of water (≈450 fs and ≈3 ps EAS components in Figures S34 and S36) reveals the ESA signature stemming from SB-CS.

The SB-CS state is forming with lifetimes of 3.1 ps and 2.7 ps, upon excitations at 500 nm and 530 nm, respectively (Figures S34 and S36a). Monitoring the decay of the SB-CS state, our global analysis, employing two distinct TA systems covering sub-ns and sub-50 ns timescales, assigns a three-exponential decay (44%, 31 ps; 33%, 2.8 ns; 17%, 9 ns), resulting in a long-lived (6%, >> 50 ns) residual signal. It is noteworthy that the SB-CS decay lifetime correlates with the recovery of the GSB (Figure S37), indicating charge recombination back to the ground state with an average lifetime of ≈2.6 ns (Table S1). While a universally agreed-upon definition for a long-lived SB-CS state is lacking, a widely accepted criterion categorizing the SB-CS as 'long-lived' is a ratio of $k_{SB\text{-}CS}/k_{CR} > 1000$.[15]

In our case, the lower limit of the $k_{SB\text{-}CS}/k_{CR}$ is 1800 (Table S2), limited by the estimation of the $\tau_4 > 50$ ns CR component, which exceeds our accessible time delays. Notably, the ratio found in BODIPY **1** in 98% of water exceeds the most common values reported in the literature for typical organic supramolecular complexes, signifying one of the highest ever reported.[16]

Cyclic voltammetry

Films of aggregates were prepared by drop casting 100 µL of a solution of aggregates (50 µM; $f_w = 98\%$) on a 0.5 cm^2 area of ITO following by drying in the dark for ~ 6 h. Prior to each experiment, the cell was purged by Ar for at least 5 min. All experiments were conducted under flowing argon. The cyclic voltammogram of aggregates showed irreversible oxidation and reduction waves at $E_{pa} = 0.50$ V and $E_{pc} = -0.21$ V, respectively.

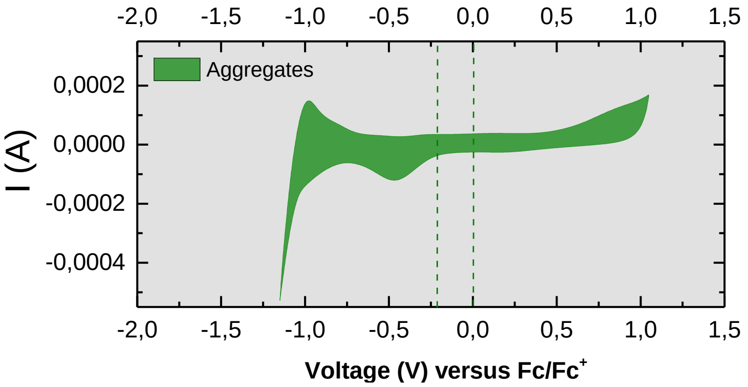

Figure S38. Cyclic voltammetry graph versus Fc/Fc+ of nanocrystalline aggregates of BODIPY **1** immobilized on transparent conductive glass electrodes coated with indium tin oxide (ITO).

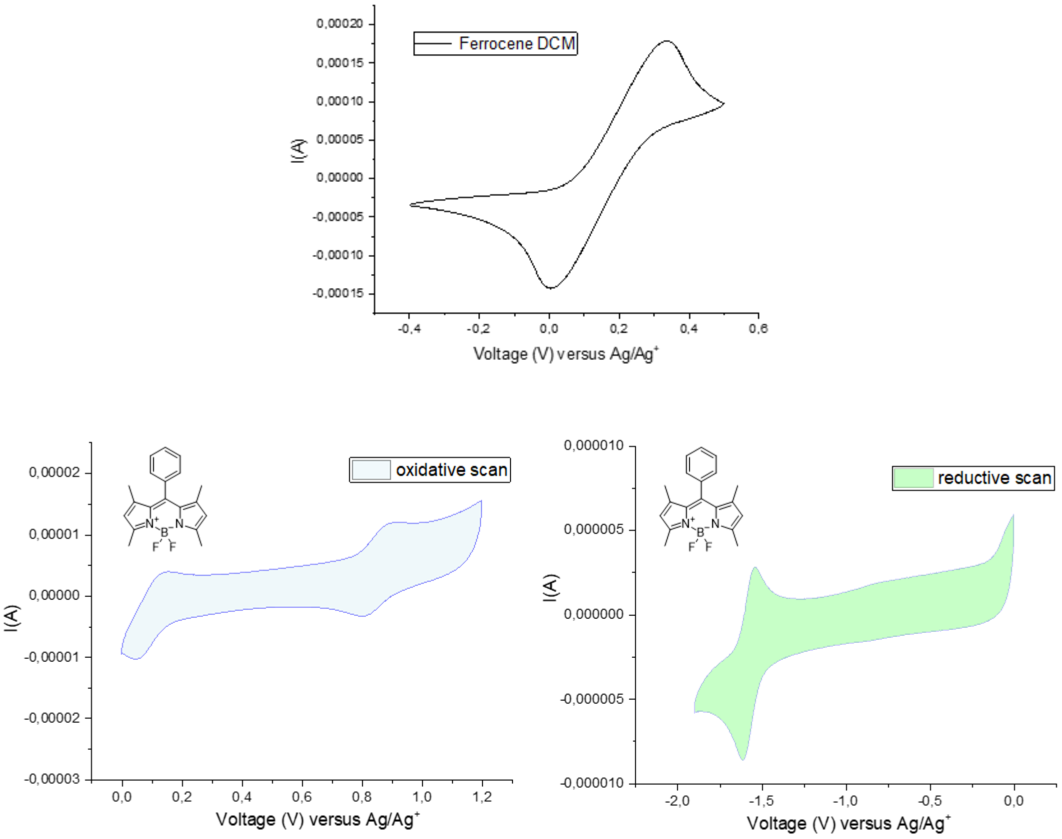

Figure S39. Cyclic voltammetry graphs versus Ag/Ag+ of BODIPY **1** in DCM. $E_{1/2}$= 0.68 V and $E_{1/2}$ = -1.75 V both reversible *vs*. F_c^+/F_c (top spectrum).

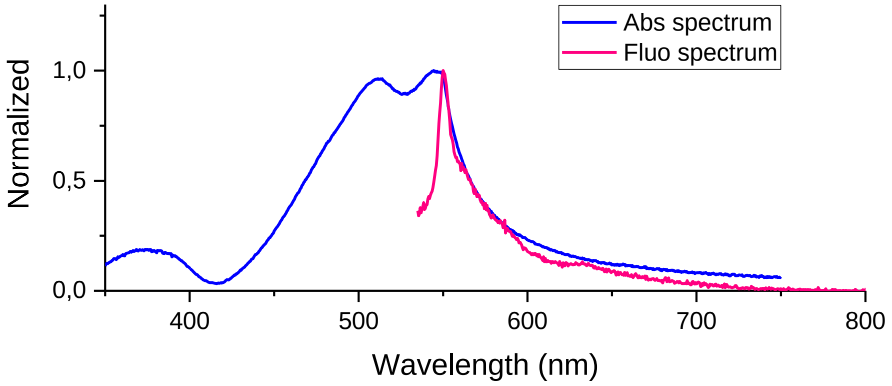

Figure S40. Optical gap (E_{S1} = 2.25 eV) obtained from the crossing point of the normalized absorption and fluorescence spectra (exc: at 510 nm) of a crystalline film of BODIPY **1** deposited onto a quartz plate from a 98% H2O - 2% MeCN solution (C_1 = 50 μM).

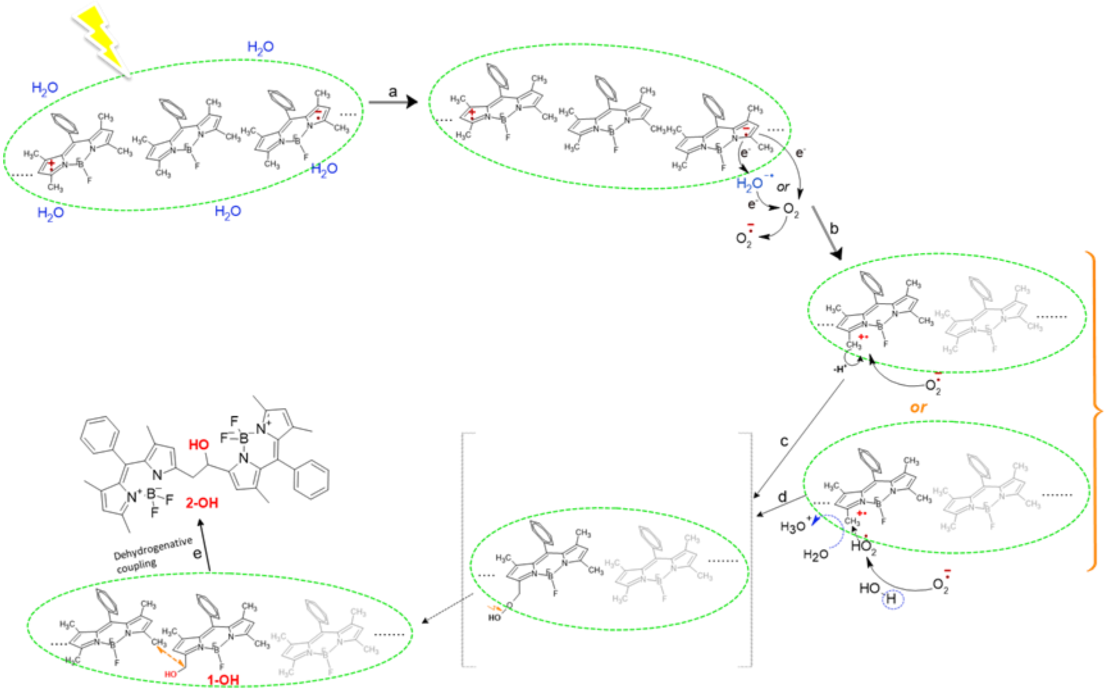

Scheme 1. Proposed mechanism for the formation of **1-OH** and **2-OH**. Full details of steps (a), (b), (c), (d), and (e) are given in the main manuscript.

The extremely low yields of the byproducts **bp1** and **bp2** prevent us from performing additional experiments to support the mechanism. However, **bp2** appears to have undergone •CH3 metathesis from a closely spaced dye to the α-position of **1**, followed by C-C cross dehydrogenative coupling. **Bp1** is likely to be an oxidized intermediate derivative in the formation of **bp2**.

Crystal Structure

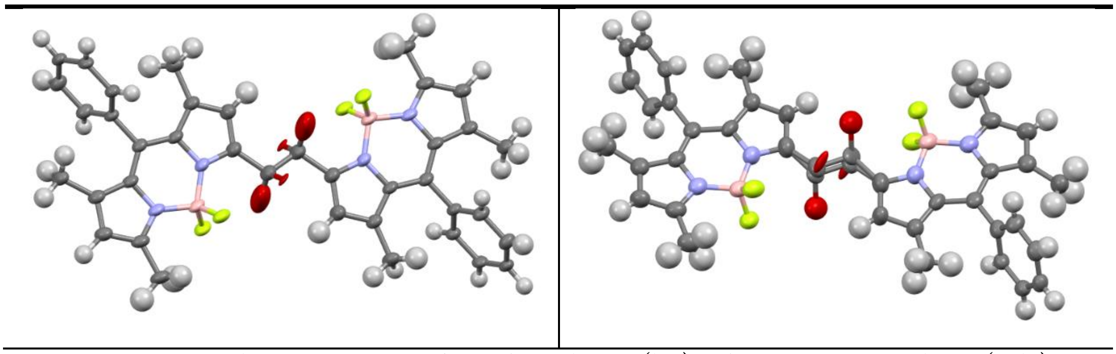

Figure S41. X-ray crystal structure of **2-OH** obtained from hexane (left) and from an aqueous solution (right).

The X-ray crystal structure obtained by crystallization from hexane shows parallel orientation of the -C(OH)-C-bridged dipyrrin cores, with their planes and transition dipole moments (μ_{eg}) being essentially coplanar and strictly parallel. Due to the presence of an equal mixture of enantiomers and packing disorder, each carbon position of the spacer exhibits one quarter occupancy for each oxygen atom.

Single crystals grown from aqueous solution of **2-OH** show a certain degree of conformational flexibility of the -C(OH)-C- core bond appearing as crystallographic disorder at the positions of the C atoms. This was not observed in the fully coplanar conformation of **2-OH** obtained from crystals grown in hexane and appears to confirm the likelihood of increased conformational flexibility of similar dimers in polar solvents.[17] The dimeric units are packed (b-axis) in a linear arrangement forming ordered, quasi-one-dimensional layers, in which *J*-dimers are themselves organized into a slip-stacked packing motif (θ_{slip}^{b} = 18.1°) with *J*-type excitonic coupling. Along the c-axis, the packing shows interleaved sets of parallel layers, in which the closest neighboring **BODIPY** rings (R = 5.33 Å) form an angle of 16.1° to each other, a rotational displacement of 40.8° and a slip angle θ_{slip}^{c} = 73^{0}.

Calculations of Excitonic Couplings

The transition dipole moment (μ_{eg}) for a given transition was calculated from the spectra using the relation of the dipole strength[#] (D);

$$D=\mu_{eg}^2=9.186x10^{-3}nf^{-2}\int\frac{\varepsilon(\tilde{v})}{v}dv \qquad (1)$$

where n is the refractive index and $f = 3n^2/(2n^2+1)$ is the local-field correction factor.

The Davydov splitting V_{ab} was calculated via equation

$$V_{ab} = 2J_c (2)$$

Where J_c stands for the strength of Coulombic interactions between transition dipoles of A and B **BODIPY** subunits and is given by the equation:

$$J_c = \frac{1}{4\pi\varepsilon_0}\frac{|\mu_A||\mu_B|}{R_{DA}^3}\kappa \qquad (3)$$

It is a function of the magnitudes of the transition dipoles μ_A, μ_B, distance R_{AB} and orientation, expressed by the κ factor given by the equation:

$$\kappa^2 = (cos\theta_{AB} - 3cos\theta_A cos\theta_B)^2 \quad (4)$$

In the above expression, θ_{AB} is the angle between the donor's emission and acceptor's absorption transition moment while θ_A andθ_B are the angles between these dipoles and the intermolecular separation vector R_{AB} joining the centroids of the interacting dipoles.

The calculated energy coupling (J_c = 226 cm^{-1}) for the strictly planar structure, using the point dipole approximation, is found to underestimate the experimentally observed (693 cm^{-1}). This is due to limitations of the method and mainly to excitonic coupling contributions from different high-energy conformers present in solution, considering that in a solvated environment local structural fluctuation can perturb the coplanar arrangement of the dipyrrin cores, thereby affecting their interchromophoric coupling.[18]

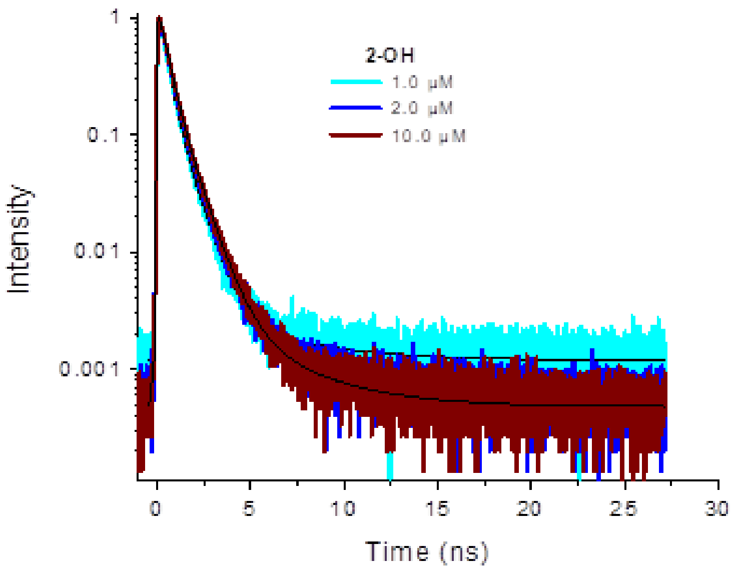

Concentration	λ_{det} (nm)	A_1	τ_1 (ns)	A_2	τ_2 (ns)	<τ> (ns)
10^{-6} M	720	0.73	0.35	0.27	0.80	0.48
$2x10^{-6}$ M	720	0.68	0.38	0.32	0.83	0.547
10^{-5} M	720	0.68	0.39	0.32	0.88	0.56

Figure S42. Nanosecond fluorescence dynamics of **2-OH** (f_w= 95%) at different concentrations.

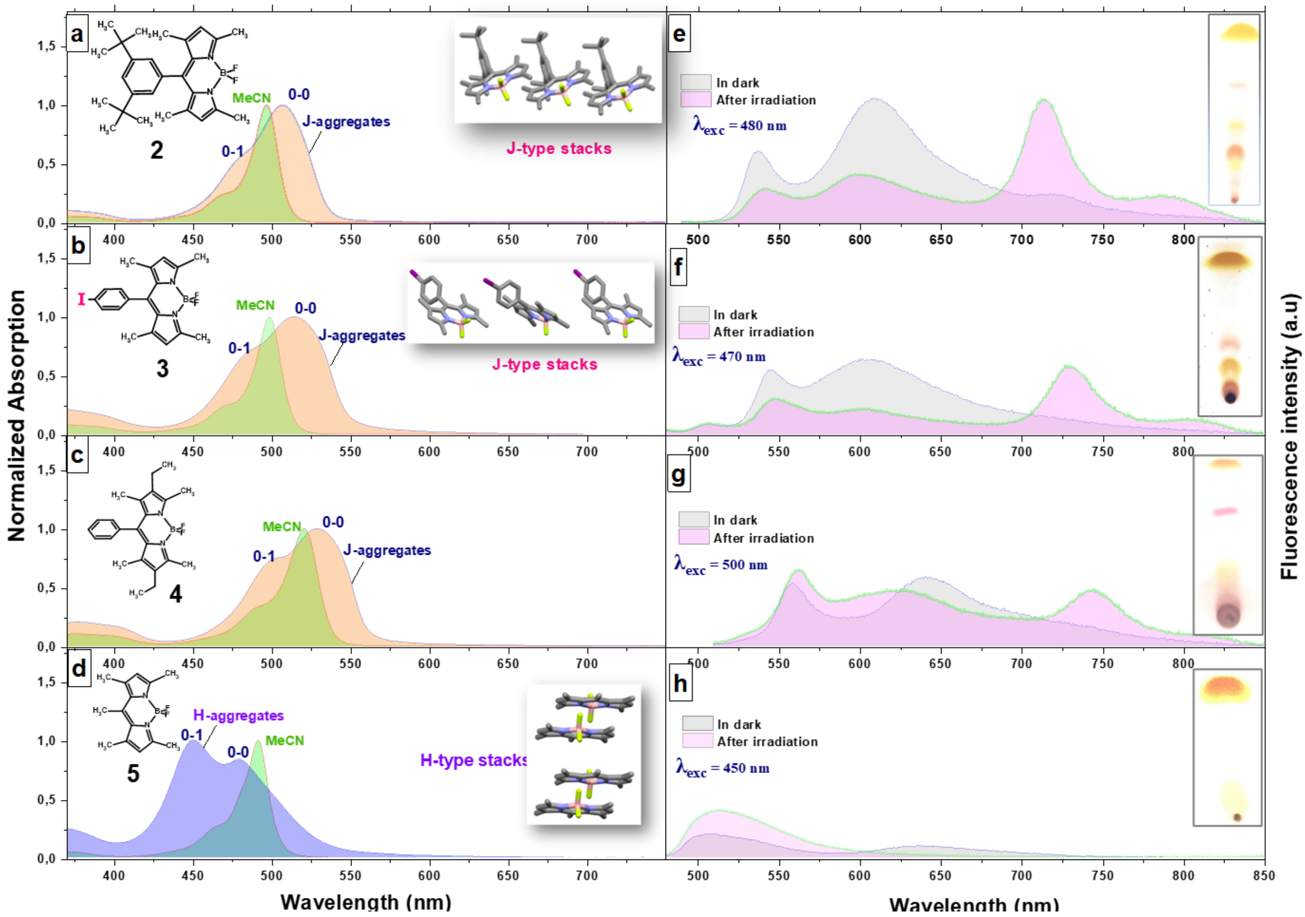

Figure S43. Normalized absorption spectra of BODIPY **2** (a)**,** **3** (b)**,** **4** (c) and **5** (d) in acetonitrile (MeCN) and of their aggregates in the presence of water (f_w = 98%; 50.0 μM). For **2**, **3**, and **4** the absorption spectra of the aggregates give a clear signature for J-type stacking (red shifted spectra with respect to the spectra of the monomers and a ratio of the 0-0 and 0-1 vibronic peaks $A_{0\text{-}0}/A_{0\text{-}1}>1$). For **5** the new absorption band to the blue of the monomer band ($A_{0\text{-}0}/A_{0\text{-}1}<1$) denotes H-type stacks (face to face arrangement of the molecules as opposed to slip-stacked motif for J-type; see ref. 41 in MS). The above spectral signatures are consistent with the corresponding stacking mode in the crystal structure (where available) of each compound, as shown in the insets. The respective fluorescence spectra of aggregates before (grey) and after (magenta) illumination (40 min, 1 mW white light LED; 3 ml sample) are shown in the right-hand panels (e), (f), (g) and (h) of the Figure S43 together with the TLC patterns. Similar behavior to BODIPY **1** was observed for the J-aggregates of **2**, **3** and **4**, suggesting that a wider range of J-aggregates of commonly used BODIPYs may also undergo such photoconversions associated with their NIR-AIE fingerprints. In contrast, the H-aggregates of 5 did not produce any remarkably detectable photoproduct, as shown by the TLC method (inset).

These observations are a further confirmation of the broad potential of BODIPY J- versus H- aggregates as a photoactive platform. It is generally observed that highly ordered *slip-stacked* packing motifs (***J-type*** *exciton coupling*) of identical dyes can facilitate the ultrafast formation and stabilization of a SB-CS state by long-range transfer of a *hole* or *electron* across the assembling dye arrays (ref: 15 in MS).

In contrast, SB-CS in *sandwich-like* molecular arrays (***H-type*** *exciton coupling*) - where the π-stacking mode dominates (cofacial stacking) - faces a bottleneck called excimer formation. Excimers or excimer-like states act as intermediate

exciton trap states that reduce the rate of SB-CS state formation; subsequently, SB-CS decays efficiently and the excitation energy dissipates to the environment, radiatively and/or non-radiatively.

References

1. Fakis, M.; Beckwith, J. S.; Seintis, K.; Martinou, E.; Nançoz, C.; Karakostas, N.; Petsalakis, I.; Pistolis, G.; Vauthey, E. Energy Transfer and Charge Separation Dynamics in Photoexcited Pyrene–BODIPY Molecular Dyads. *Phys. Chem. Chem. Phys.* **2018**, 20 (2), 837–849. https://doi.org/10.1039/C7CP06914F.

2. Kaloudi-Chantzea, A.; Martinou, E.; Seintis, K.; Karakostas, N.; Giastas. P.; Pitterl, F.; Oberacher, H.; Fakis M.; Pistolis G. Formation of a highly-ordered rigid multichromophoric 3D supramolecular network by combining ionic and coordination-driven self-assembly. *Chem. Commun.* **2016**, 52, 3388-3391. https://doi.org/10.1039/C5CC10335E.

3. Sun, Y. 4,4-Difluoro-8-(4-iodophenyl)-1,3,5,7-tetramethyl-3a-aza-4a-azonia-4-borata-s-indacene. *Acta Cryst.* **2012**, E68, o1302. doi:10.1107/S1600536812004072.

4. Gabe, Y.; Urano, Y.; Kikuchi, K.; Kojima H.; Nagano, T. Highly Sensitive Fluorescence Probes for Nitric Oxide Based on Boron Dipyrromethene ChromophoreRational Design of Potentially Useful Bioimaging Fluorescence Probe. *J. Am. Chem. Soc.* **2004**, 126, 3357-3367. https://doi.org/10.1021/ja037944j.

5. Marfin, Y. S.; Banakova, E. A.; Merkushev, D. A.; Usoltsev, S. D.; Churakov A. V. Effects of Concentration on Aggregation of BODIPY-Based Fluorescent Dyes Solution. *J. Fluoresc.* **2020** 30, 1611–1621. https://doi.org/10.1007/s10895-020-02622-y.

6. Stoll, S.; Schweiger, A. EasySpin, a Comprehensive Software Package for Spectral Simulation and Analysis in EPR. *J. Magn. Reson.* **2006**, 178 (1), 42–55. https://doi.org/10.1016/j.jmr.2005.08.013.

7. Kabsch, W. XDS. *Acta Cryst. D*66 **2010**, 125-132. https://doi.org/10.1107/S0907444909047337.

8. Sheldrick, G. M. A short history of SHELX. *Acta Cryst. A*64 **2008,** 112–122. https://doi.org/10.1107/S0108767307043930.

9. Brouwer, A. M. Standards for photoluminescence quantum yield measurements in solution (IUPAC Technical Report). *Pure Appl. Chem.* **2011**, 83 (12), 2213–2228. https://doi.org/10.1351/PAC-REP-10-09-31.

10. Karlsson, J. K. G.; Woodford, O. J.; Mustroph, H.; Harriman, A. Cyanine Dyes as Ratiometric Fluorescence Standards for the Far-Red Spectral Region. *Photochem. Photobiol. Sci.* **2018**, 17 (1), 99–106. https://doi.org/10.1039/C7PP00333A.

11. Cerullo, G.; Manzoni, C.; Lüer, L.; Polli, D. Time-Resolved Methods in Biophysics. 4. Broadband Pump—Probe Spectroscopy System with Sub-20 Fs Temporal Resolution for the Study of Energy

Transfer Processes in Photosynthesis. *Photochem. Photobiol.* **2007**, 6 (2), 135–144. https://doi.org/10.1039/B606949E.

12. Snellenburg, J. J.; Laptenok, S.; Seger, R.; Mullen, K. M.; Stokkum, I. H. M. van. Glotaran: A Java-Based Graphical User Interface for the R Package TIMP. *J. Stat. Softw.* **2012**, 49, 1–22. https://doi.org/10.18637/jss.v049.i03.

13. Buettner G. R. Spin trapping: ESR parameters of spin adducts. *Free Radic. Biol. Med.* **1987**, 3 (4), 259-303. doi: 10.1016/s0891-5849(87)80033-3. PMID: 2826304.

14. Estergreen, L.; Mencke, A. R.; Cotton, D. E.; Korovina, N. V.; Michl, J.; Roberts, S. T.; Thompson, M. E.; Bradforth, S. E. Controlling Symmetry Breaking Charge Transfer in BODIPY Pairs. *Acc. Chem. Res.* **2022**, *55* (11), 1561–1572. https://doi.org/10.1021/acs.accounts.2c00044.

15. Hou, Y.; Zhang, X.; Chen, K.; Liu, D.; Wang, Z.; Liu, Q.; Zhao, J.; Barbon, A. Charge Separation, Charge Recombination, Long-Lived Charge Transfer State Formation and Intersystem Crossing in Organic Electron Donor/Acceptor Dyads. *J Mater Chem C Mater* **2019**, 7 (39), 12048–12074. https://doi.org/10.1039/C9TC04285G.

16. Sebastian, E.; Hariharan, M. Symmetry-Breaking Charge Separation in Molecular Constructs for Efficient Light Energy Conversion. *ACS Energy Lett.* **2022**, 7 (2), 696–711.

17. Patalag, L. J.; Ho, L. P.; Jones, P. G.; Werz, D. B. Ethylene-Bridged Oligo-BODIPYs: Access to Intramolecular J-Aggregates and Superfluorophores. *J. Am. Chem. Soc.* **2017**, *139* (42), 15104–15113. https://doi.org/10.1021/jacs.7b08176.

18. Alden, R. G.; Johnson, E.; Nagarajan, V.; Parson, W. W.; Law C. J.; Cogdell, R. G. Calculations of Spectroscopic Properties of the LH2 Bacteriochlorophyll–Protein Antenna Complex from Rhodopseudomonas acidophila. *J. Phys. Chem. B* **1997**, 101,23, 4667 - 4680. https://doi.org/10.1021/jp970005r.
